# Telephone-Based Rehabilitation Intervention to Optimize Activity Participation After Breast Cancer: A Randomized Clinical Trial

**DOI:** 10.1001/jamanetworkopen.2024.2478

**Published:** 2024-03-22

**Authors:** Kathleen Doyle Lyons, Stephen B. Wechsler, Deborah B. Ejem, Courtney J. Stevens, Andres Azuero, Sarah Khalidi, Mark T. Hegel, Sarah M. dos Anjos, Megan E. Codini, Mary D. Chamberlin, Jamme L. Morency, Jazmine Coffee-Dunning, Karen E. Thorp, Danielle Z. Cloyd, Susan Goedeken, Robin Newman, Colleen Muse, Gabrielle Rocque, Kimberly Keene, Maria Pisu, Jennifer Echols, Marie A. Bakitas

**Affiliations:** 1 Department of Occupational Therapy, Massachusetts General Hospital Institute of Health Professions, Boston; 2 School of Nursing, University of Alabama at Birmingham, Birmingham; 3 Department of Psychiatry, Geisel School of Medicine at Dartmouth, Dartmouth College, Hanover, New Hampshire; 4 School of Health Professions, Department of Occupational Therapy, University of Alabama at Birmingham, Birmingham; 5 Department of Rehabilitation, Berkshire Medical Center, Pittsfield, Massachusetts; 6 Dartmouth Cancer Center, Geisel School of Medicine at Dartmouth, Lebanon, New Hampshire; 7 Rehabilitation Medicine, Dartmouth Health, Lebanon, New Hampshire; 8 Department of Neurology, Mass General Brigham, Boston, Massachusetts; 9 Department of Occupational Therapy, Sargent College of Health and Rehabilitation Sciences, Boston University, Boston, Massachusetts; 10 Department of Medicine, Division of Hematology and Oncology, Heersink School of Medicine, University of Alabama at Birmingham, Birmingham; 11 Department of Medicine, Division of Gerontology, Geriatrics, and Palliative Care, Heersink School of Medicine, University of Alabama at Birmingham, Birmingham; 12 Center for Palliative and Supportive Care, Heersink School of Medicine, University of Alabama at Birmingham, Birmingham; 13 O’Neal Comprehensive Cancer Center, University of Alabama at Birmingham, Birmingham; 14 Department of Radiation Oncology, Heersink School of Medicine, University of Alabama at Birmingham, Birmingham; 15 Division of Preventive Medicine, Heersink School of Medicine, University of Alabama at Birmingham, Birmingham

## Abstract

**Importance:**

Following treatment, breast cancer survivors face challenges participating in valued activities.

**Objective:**

To determine whether a telephone-based coaching rehabilitation intervention enhances activity participation in the year following breast cancer treatment.

**Design, Setting, and Participants:**

In this multisite, single-blind randomized clinical trial (Optimizing Functional Recovery of Breast Cancer Survivors), recruitment occurred between August 28, 2019, and April 30, 2022. Data collection was completed by April 1, 2023. Participants were recruited from 2 cancer centers (Dartmouth College and the University of Alabama at Birmingham) and via social media advertisements. Women aged 18 years or older who had completed primary treatment for stage I to III breast cancer within 1 year and reported participation restrictions were eligible to participate. Randomization was stratified by site, treatment, and time since treatment.

**Interventions:**

The intervention, delivered via telephone over 9 sessions, used behavioral activation and problem-solving principles to promote activity participation. The education-based attention control condition was delivered via telephone at matched intervals.

**Main Outcomes and Measures:**

The primary outcome was participation, assessed using 5 measures, including Patient-Reported Outcomes Measurement Information System (PROMIS) social participation-satisfaction measure. One individualized outcome allowed participants to specify activities for which they wanted to foster recovery. Outcomes were collected by telephone by blinded coordinators at baseline and at 8, 20, and 44 weeks. The individualized outcome was assessed at the first and last intervention and control session.

**Results:**

Among 1996 patients identified, 303 were eligible and enrolled. Of these, 284 women (94%; mean [SD] age, 56.1 [10.2] years) completed baseline assessments and were randomized, and 81% or more of each group completed the final assessment with no adverse events. Of those who completed the final assessment, 118 of 144 (82%) were in the intervention group, and 113 of 140 (81%) were attention control participants. Between-group differences were not statistically significant for the main measures of PROMIS satisfaction (week 20: Cohen *d*, 0.1 [95% CI, −0.09 to 0.29] and week 44: Cohen *d*, −0.08 [95% CI, −0.27 to 0.11]) and ability (week 20: Cohen *d*, 0.15 [95% CI, −0.06 to 0.37] and week 44: Cohen *d*, −0.08 [95% CI, −0.27 to 0.11]). On the individualized outcome, intervention participants reported significantly greater improvements in activity satisfaction (Cohen *d*, 0.76 [95% CI, 0.48-1.02]) and performance (Cohen *d*, 0.60 [95% CI, 0.32-0.87]).

**Conclusions and Relevance:**

In this randomized clinical trial, the intervention catalyzed greater improvements in self-selected activity participation and goal disengagement but did not otherwise accelerate recovery compared with the control condition. Future research should determine what intervention features may lead to the greatest reductions in participation restrictions and other measures that may detect functional recovery.

**Trial Registration:**

ClinicalTrials.gov Identifier: NCT03915548

## Key Points

**Question:**

Compared with an attention control condition, what is the effect of a behavioral activation and problem-solving intervention on participation restrictions among breast cancer survivors in the year following treatment?

**Findings:**

In this randomized clinical trial that included 284 women, the intervention resulted in significantly greater improvements in self-selected activity participation but did not otherwise accelerate recovery compared with the control condition.

**Meaning:**

These results suggest that research is needed to determine whether time or the conditions’ common features (eg, support, individualized attention to elicit priorities, and education) led to participation improvements or whether objective measures could better illuminate the intervention’s effect on functional recovery.

## Teaser

This randomized clinical trial assesses the effect of a behavioral activation and problem-solving intervention compared with an attention control condition on participation restrictions among US breast cancer survivors in the year following treatment.

## Introduction

Participation restrictions (difficulties people face when fulfilling life roles and performing valued activities^1^) are more prevalent among cancer survivors compared with healthy peers; nearly one-third of cancer survivors report restrictions.^2^ Restrictions in work, recreational, and daily activities can persist 2 years^3^ to 6 years^4^ after breast cancer treatment.^5,6,7,8,9^ Full recovery from cancer includes the ability to resume and enjoy activities that are needed to take care of oneself and one’s family and fully contribute to and participate in society.^10,11^

Absent evidence-based interventions to mitigate participation restrictions,^12,13^ our group developed and pilot tested^14,15^ a telephone-based coaching intervention that integrated rehabilitation principles^16^ and behavioral therapies^17,18^ to promote activity participation among breast cancer survivors. The intervention, behavioral activation and problem-solving (BA/PS), aimed to help women adapt activities, modify environments, and develop weekly goals and action plans that promoted successful activity participation. We conducted a randomized clinical trial (RCT) testing whether BA/PS is efficacious in enhancing participation (primary outcome), quality of life (QOL) (secondary outcome), and adaptive coping, goal adjustment, and reducing distress (exploratory outcomes) among women who had completed primary breast cancer treatment within the past year. We hypothesized that BA/PS would outperform an attention control condition on these outcomes.^19^

## Methods

### Design

This was a single-blind RCT (Optimizing Functional Recovery of Breast Cancer Survivors) with 1:1 allocation to 2 arms. The trial protocol and analytic plan (Supplement 1) were approved by the investigators’ institutional review boards (Dartmouth College, University of Alabama at Brimingham, Dartmouth-Hitchcock Health, and Massachusetts General Hospital). Participants were contacted by telephone to assess interest, screen for eligibility, and initiate informed consent, which was documented in writing. This study followed the Consolidated Standards of Reporting Trials (CONSORT) reporting guideline for randomized clinical trials.^20^

### Eligibility Criteria

Inclusion criteria included being a woman aged 18 years or older; having reduced participation (ie, scoring ≥10 on the Work and Social Adjustment Scale^21^); and having a diagnosis of stage I to III breast cancer, having no disease recurrence, and being within 1 year of completing locoregional treatment and/or chemotherapy with curative intent. The Work and Social Adjustment Scale uses self-ratings of level of impairment, ranging from 0 (no impairment) to 8 (very severe impairment) within 5 domains: work, home management, social leisure, private leisure, and relationships. A score of 10 or more indicates either a modest level of impairment affecting most domains or a moderate to severe impairment affecting few domains. Exclusions included being non-English speaking, having noncorrectable hearing loss; having moderate to severe cognitive impairment (ie, a score <3 on a 6-item cognitive screener^22^); and having a documented or self-reported history of severe mental illness, current major depressive disorder, suicidal ideation, or substance use.

### Setting and Recruitment

Recruitment occurred between August 28, 2019, and April 30, 2022; processes are detailed elsewhere.^23^ Data collection was completed by April 1, 2023. We initially recruited women from the cancer centers at Dartmouth College and the University of Alabama at Birmingham. When the COVID-19 pandemic restrictions limited in-person recruitment, we mailed letters to clinic patients and launched a Social media advertising campaign. Participants were told that we were comparing 2 ways to foster functional recovery after breast cancer treatment: goal setting and problem-solving vs education about survivorship topics.

### Randomization

The statistician (A.A.) created a computer-generated 1:1 randomization scheme, stratified by site (Dartmouth College vs the University of Alabama at Birmingham), treatment (chemotherapy vs no chemotherapy), and time since treatment completion (<6 months or 6-12 months) and blocked within strata (block lengths of 2 and 4 varied randomly). After baseline survey completion, coordinators notified the principal investigator (PI; K.D.L.) and project manager (J.C.-D.) who revealed the randomization assignment to the coach. The coach contacted the participant, revealed the assignment, and scheduled the intervention sessions.

### Conditions

#### Dose and Delivery

Nine occupational therapist coaches (S.M.d.A., M.E.C., J.L.M., K.E.T., D.Z.C., S.G., R.N., C.M., and J.E.) delivered the 9 BA/PS intervention sessions; 8 of those occupational therapists and 1 nurse delivered the 9 attention control sessions. Both interventions were delivered by telephone. The first 6 sessions occurred weekly, and the last 3 sessions occurred monthly. Each condition is described briefly and detailed elsewhere.^19,24^

#### Behavioral Activation and Problem-Solving

Participants were mailed a workbook containing education regarding the intervention purpose, how activity engagement can promote well-being and recovery, the importance of a positive problem orientation when tackling challenges, and the principles of modifying the “who, when, where, and how” of activity engagement to maximize energy, ease, and enjoyment. In each session, coaches guided BA/PS participants to use a goal-setting, problem-solving, and action-planning worksheet to plan at least 1 self-selected activity to perform in the coming week.

#### Attention Control

The attention control condition provided self-management educational content, drawn from the Springboard Beyond Cancer website that was codeveloped by the National Cancer Institute and the American Cancer Society.^25^ Participants were provided a link to the website or a PDF of each topic if desired. Weekly educational topics were participant selected to mimic the individualized nature of the BA/PS condition. Sessions consisted of 4 steps: (1) review the previous week, (2) review the education topic, (3) discuss the degree to which the information resonates with the participant’s experience, and (4) identify the next session’s topic.

#### Training and Fidelity Monitoring

The PI trained coaches during a full-day session using instruction, demonstration, and role-playing. Practice sessions were recorded, and the PI reviewed them and provided feedback. Each coach performed at least 5 practice BA/PS sessions with fidelity before being assigned a participant. The PI and coaches met weekly for supervision.

Each telephone session was audio-recorded with participant permission. We randomly sampled 2 sessions from 15% of participants in each condition and assessed fidelity to the protocol using dichotomous ratings of prescribed and proscribed behaviors and continuous ratings of delivery quality. Results were discussed with coaches during weekly supervision. Protocol fidelity was excellent (≥4.6 on a 5-point scale); details are presented elsewhere.^24^

### Data Collection

#### Administration and Schedule

Coaches recorded participants’ individual activity participation targets (using the Canadian Occupational Performance Measure [COPM]^26^) in the first (baseline) and last (approximately 20 weeks) BA/PS and attention control sessions. Scores for all other COPM scores were collected at baseline, 8, 20, and 44 weeks by telephone by trained coordinators blinded to group assignment. Participants could complete measures electronically, if needed. Participants received $25 after completing each of the first 3 assessments and $30 after completing the last assessment. All measures were prespecified prior to RCT commencement.^19^

#### Sociodemographic and Clinical Characteristics

To characterize the sample, participants self-reported their age, race and ethnicity (using investigator-provided categories), employment status, educational level, marital status, insurance status, number of dependent children living at home, household income, cancer treatment and stage, rurality, and comorbidities.^27^ Race and ethnicity categories included Asian, Black or African American, Hispanic or Latina, non-Hispanic or non-Latina, White, and other race (the category was selected without providing more information).

#### Participation Satisfaction and Ability, Productivity, and Individual Activity Targets

The primary outcome was participation. Absent a gold standard measure,^28^ we used multiple measures to triangulate participation restrictions. The primary measure of participation was the Patient-Reported Outcomes Measurement Information System (PROMIS) Short Form, version 2.0 (Satisfaction with Social Roles and Activities 8a^29,30^), which assessed satisfaction with daily routines and activities.^31^ The PROMIS Short Form, version 2.0 (Ability to Participate in Social Roles and Activities 8a^29,30^) measured self-reported ability. T scores ranged from 26.2 to 65.6 on a scale of 0 to 100, with higher scores indicating greater satisfaction with participation in social roles and activities. These 8-item scales, which have previously been used in cancer survivors,^32^ addressed routine, work, leisure, family, and social activities. We assumed a minimal important difference of approximately 3.9 for the T scores, approximately 10% of the practical range of the scale (4 SD).^33,34^ The disability days section of the Medical Expenditure Panel Survey^35,36^ captured days missed from work and lost household productivity due to physical illness, injury, or mental or emotional problems. The Work Limitations Questionnaire^37^ assessed productivity with an overall score indicating the percentage decrement in productivity in the previous 2 weeks across 4 subscales (time, physical, mental or interpersonal, and output). Finally, the COPM, an individualized outcome measure, allowed participants to specify activities for which they wanted to foster recovery.^26^ Participants rated up to 5 self-selected activities using Likert scales (ranging from 1 to 10) for 3 characteristics: importance, performance, and satisfaction. Higher scores indicated greater participation.

#### Quality of Life

The Functional Assessment of Cancer Therapy-General (FACT-G)^38^ is a 27-item questionnaire with 4 subscales: physical, social, emotional, and functional well-being. A 5-point increase in overall score indicates meaningful improvement.^39^

#### Adaptive Coping, Goal Adjustment, and Distress

The Brief-COPE inventory^40^ assessed participants’ use of active coping, planning, and positive reframing, which were outcomes that our group’s pilot study indicated were increased by BA/PS.^14^ The 10-item Goal Disengagement and Goal Reengagement Scale^41^ includes 2 subscales measuring dispositional goal disengagement (4 items; ie, an inclination to relinquish untenable goals) and reengagement (6 items; ie, an inclination to commit to new goals). Distress was assessed using the Hospital Anxiety and Depression Scale,^42^ a 14-item self-report measure of depressive and anxious symptoms.

### Statistical Analysis

#### Sample Size

Target recruitment was approximately 300 participants, with expected 20% attrition and complete data on 240 participants. At a corrected significance level of 0.05 / 3.00 = 0.017, with 3 follow-up measurements and intraparticipant correlation of 0.5, a sample size of n = 120 per arm provided 80% power to detect a standardized time-averaged difference between conditions (Cohen *d*, 0.35), which would be slightly smaller than the minimal important difference of  3.9 for the primary outcome of participation measured by the PROMIS scales.

#### Data Analysis

We conducted all analyses following an intention-to-treat principle. We examined balance between study arms related to baseline characteristics and baseline outcomes using descriptive statistics and measures of effect size (eg, Cohen *d* [small, 0.2; medium, 0.5; and large, 0.8] and Cramer *V* [small, 0.1; medium, 0.3; and large,  0.5]).^43^ We examined associations of baseline data with completion of data collection using measures of effect size and bivariate tests of association. Characteristics with a nontrivial magnitude of association with incomplete data (ie, Cohen *d*, ≥0.25 or Cramer *V*, ≥0.2) were used as controlling covariates in subsequent analyses to reduce potential bias caused by missing data.^44^

Generalized linear mixed-effect models with participant-specific random effects were fitted to estimate between-group mean contrasts at follow-up time points, using baseline values, indicator variables for time, study group, time-by-group interaction, and covariates associated with missing data as fixed effects. The models used all data at each time point. A Poisson distribution model with a log link was used for disability days, resulting in between-group contrasts as mean ratios, which served as measures of effect size. Models for continuous outcomes used the gaussian distribution, resulting in between-group contrasts as mean differences. We used baseline data to estimate a pooled SD for each continuous outcome and standardized between-group contrasts as effect sizes (Cohen *d*). We used a multiple degrees of freedom test for the time-by-group interaction terms as an overall test of between-group differences in a longitudinal outcome trajectory. We used a false discovery rate,^45^ applied separately to primary and exploratory outcomes, to control for multiple inferences. We first applied a correction to the tests of between-group comparisons in a trajectory (a multiple degrees of freedom test for each outcome), and if a significant difference in trajectories was found, we applied a correction to the between-group contrasts at each follow-up time point. The significance level for the multiplicity-adjusted *P* values was set at .05 and was 2-sided. An additional model was used to estimate between-group contrasts in COPM scores between the first and last sessions. Analyses were conducted using R, version 4.2.3 (R Project for Statistical Computing).^46^

## Results

### Participants

Enrollment occurred from August 28, 2019, and April 30, 2022, 3 months longer than planned. Intervention delivery ended November 4, 2022, and data collection ended on April 1, 2023. The CONSORT diagram (Figure) depicts participant flow through the study. Among 1996 women identified, 303 enrolled, and of those, 284 (94%; mean [SD] age, 56.1 [10.2] years) completed baseline assessments and were randomized. Of those who completed the final assessment, 118 of 144 (82%) were BA/PS participants, and 113 of 140 (81%) were attention control participants.

**Figure.**
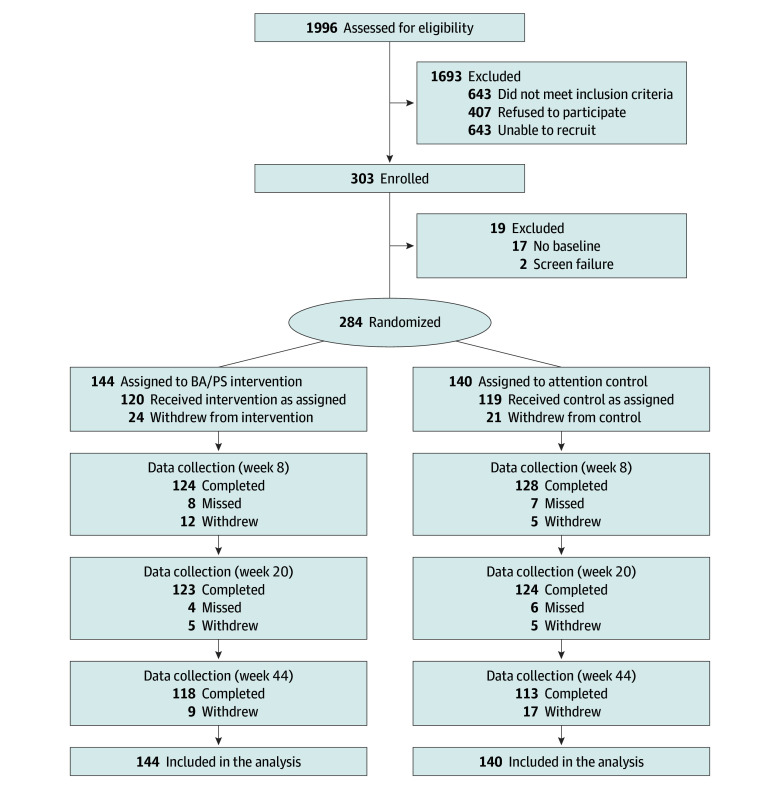
Participant Flow Data collection for week 8 was the follow-up 1 time point; week 20, follow-up 2; and week 44, follow-up 3. BA/PS indicates behavioral activation and problem-solving.

Treatment retention was high; of 144 women randomized to BA/PS, 127 (88%) completed at least the 6 weekly sessions, and 120 (83%) completed all 9 sessions. Of the 140 women randomized to the attention control condition, 126 (90%) completed at least the 6 weekly sessions, and 119 (85%) completed all 9 sessions. Participants reported no adverse events.

### Baseline Characteristics

Participant characteristics and baseline survey scores (Table 1) were similar, except for a mild imbalance in the goal adjustment scale’s disengagement score (Cohen *d*, 0.27). Of the total participants, 2 (0.7%) were Asian; 47 (16.5%), Black or African American; 6 (2.1%), Hispanic or Latina; 278 (97.9%), non-Hispanic or non-Latina; 234 (82.4%), White; and 1 (0.4%), other race. Among participants, 190 (66.9%) had a college degree, and most (187 [65.8%]) were married or partnered. Most participants (232 [81.7%]) had stage I or II cancer and received radiation (218 [76.8%]) and/or chemotherapy (186 [65.5%]), and over half (163 [57.4%]) completed treatment within 6 months of enrollment. Mean (SD) baseline PROMIS satisfaction T scores were 45.7 (6.7), and ability T scores were 43.3 (5.3), which were 0.5 and 0.7 SDs lower than those for the general adult population, respectively. Participants showed, on average, lower QOL compared with the general population^47^anxiety symptoms.^42^

**Table 1.** Participant Characteristics Overall and by Study Arm

Characteristic	Participants, No. (%)			Effect size^a^
	All (N = 284)	BA/PS intervention (n = 144)	Attention control (n = 140)	
Age, mean (SD), y	56.1 (10.2)	56.0 (9.9)	56.2 (10.6)	0.02
Recruitment site				
Dartmouth Cancer Center	71 (25.0)	35 (24.3)	36 (25.7)	0.02
Social media	157 (55.3)	80 (55.6)	77 (55.0)	
UAB clinic	56 (19.7)	29 (20.1)	27 (19.3)	
Race				
Asian	2 (0.7)	1 (0.7)	1 (0.7)	0.12
Black or African American	47 (16.5)	18 (12.5)	29 (20.7)	
White	234 (82.4)	124 (86.1)	110 (78.6)	
Other^b^	1 (0.4)	1 (0.7)	0	
Ethnicity				
Hispanic or Latina	6 (2.1)	4 (2.8)	2 (1.4)	0.05
Non-Hispanic or non-Latina	278 (97.9)	140 (97.2)	138 (98.6)	
Employment status				
Employed (full-time or part-time)	152 (53.5)	75 (52.1)	77 (55)	0.06
Homemaker or retired	77 (27.1)	42 (29.2)	35 (25.0)	
On short-term or long-term disability	25 (8.9)	12 (8.3)	13 (9.3)	
Unemployed	22 (7.7)	10 (6.9)	12 (8.6)	
Other	6 (2.1)	3 (2.1)	3 (2.1)	
Missing	2 (0.7)	2 (1.4)	0	
Educational level				
Some high school/High school graduate	27 (9.5)	9 (6.3)	18 (12.9)	0.11
Some college (2 y)	65 (22.9)	35 (24.3)	30 (21.4)	
Graduate (4 y, master’s degree, or doctorate)	190 (66.9)	98 (68.1)	92 (65.7)	
Missing	2 (0.7)	2 (1.3)	0	
Marital status				
Never married	19 (6.7)	8 (5.6)	11 (7.9)	0.07
Married or living with a partner	187 (65.8)	98 (68.1)	89 (63.6)	
Separated or divorced	65 (22.9)	32 (22.2)	33 (23.6)	
Widowed	12 (4.2)	5 (3.5)	7 (5.0)	
Missing	1 (0.3)	1 (0.6)	7 (5)	
No. of dependent children living at home				
0	198 (69.7)	103 (71.5)	95 (67.9)	0.08
1	42 (14.8)	21 (14.6)	21 (15.0)	
2	31 (10.9)	15 (10.4)	16 (11.4)	
3	9 (3.2)	3 (2.1)	6 (4.3)	
4	3 (1.0)	1 (0.7)	2 (1.4)	
Missing	1 (0.4)	1 (0.7)	0	
Insurance status				
Other or none or self-pay	12 (4.2)	4 (2.8)	8 (5.7)	0.09
Medicaid or Medicare or other government insurance	78 (27.5)	37 (25.7)	41 (29.3)	
Private through employer	181 (63.7)	96 (66.7)	85 (60.7)	
Self-purchased	13 (4.6)	7 (4.9)	6 (4.3)	
Household income per y				
<$40 000	60 (21.1)	28 (19.4)	32 (22.9)	0.06
≥$40 000	220 (77.5)	114 (79.2)	106 (75.7)	
Missing	4 (1.4)	2 (1.4)	2 (1.4)	
Rurality				
Urban	181 (63.7)	95 (66.0)	86 (61.4)	0.05
Rural	93 (32.8)	44 (30.5)	49 (35.0)	
Missing	10 (3.5)	5 (3.5)	5 (3.6)	
Cancer stage				
I	119 (41.9)	55 (38.2)	64 (45.7)	0.08
II	113 (39.8)	60 (41.7)	53 (37.9)	
III	52 (18.3)	29 (20.1)	23 (16.4)	
Treatment				
Surgery	282 (99.3)	143 (99.3)	139 (99.3)	0
Radiation	218 (76.8)	118 (81.9)	100 (71.4)	0.12
Chemotherapy	186 (65.5)	96 (66.7)	90 (64.3)	0.03
Time since end of primary treatment, mo				
<6	163 (57.4)	82 (56.9)	81 (57.9)	0.01
6-12	121 (42.6)	62 (43.1)	59 (42.1)	
Disability days, mean (SD)				
Days missed work^c^	2.5 (5.3)	3.0 (6.0)	2.1 (4.5)	0.17
Days in bed	3.5 (6.4)	2.8 (5.3)	4.2 (7.3)	0.22
PROMIS T score^d^				
Satisfaction with social roles and activities	45.7 (6.7)	45.6 (6.5)	45.8 (6.9)	0.02
Ability to participate in social roles and activities	43.3 (5.3)	43.4 (5.2)	43.3 (5.5)	0.02
WLQ scale scores^c^^,^^e^	35.3 (18.3)	34.5 (17.4)	36.0 (19.3)	0.08
FACT-G score^f^				
Physical	17.0 (5.3)	17 (5.5)	17 (5.2)	0.01
Social	19.5 (5.9)	19.9 (5.2)	19.1 (6.5)	0.14
Emotional	15.9 (4.6)	15.9 (4.5)	15.8 (4.6)	0.02
Functional	16.4 (5.3)	16.5 (5.3)	16.2 (5.3)	0.06
Overall score	68.7 (16.9)	69.3 (16.3)	68.1 (17.5)	0.07
Brief-COPE inventory scores^g^				
Active coping	6.4 (1.6)	6.3 (1.5)	6.5 (1.6)	0.13
Planning	6.4 (1.7)	6.3 (1.7)	6.6 (1.6)	0.19
Positive reframing	6.4 (1.7)	6.4 (1.7)	6.6 (1.7)	0.15
GDGRS scores				
Goal disengagement^h^	10.4 (3.1)	10 (2.9)	10.9 (3.3)	0.27
Goal reengagement^i^	21.3 (3.9)	21.5 (3.8)	21.2 (3.9)	0.07
HADS scores^j^				
Anxiety	9.2 (4.3)	9.4 (4.3)	9.0 (4.3)	0.08
Depression	6.3 (3.8)	6.0 (3.5)	6.7 (4.1)	0.17
Work and Social Adjustment Scale score^k^	16.9 (6.8)	16.3 (6.5)	17.5 (7.1)	0.18

Abbreviations: BA/PS, behavioral activation and problem-solving; FACT-G, Functional Assessment of Cancer Therapy-General; GDGRS, Goal Disengagement and Goal Reengagement Scale; HADS, Hospital Anxiety and Depression Scale; PROMIS, Patient-Reported Outcomes Measurement Information System; UAB, University of Alabama at Birmingham; WLQ, Work Limitations Questionnaire.

^a^ Effect size is presented as Cohen *d* for age, disability days, and all questionnaires and scales. Effect size is presented as Cramer *V* for recruitment site, race, ethnicity, employment status, educational level, marital status, insurance status, household income, rurality, cancer state, treatment, and time since end of primary treatment.

^b^ Other race was selected without providing more information.

^c^ Only currently employed (n = 152).

^d^ T scores range from 0 to 100, with higher scores indicating greater satisfaction and ability to participate in social roles and activities.

^e^ Scores range from 0% to 100%, with higher scores indicating greater decrement in productivity.

^f^ A 27-item questionnaire with 4 subscales: physical, social, emotional, and functional well-being. A 5-point increase in overall score indicates meaningful improvement. Scores range from 0 to 108, with higher scores indicating greater quality of life.

^g^ Scores range from 0 to 6, with higher scores indicating greater use of the coping strategy.

^h^ Scores range from 4 to 20, with higher scores indicating greater tendency to goal disengagement.

^i^ Scores range from 6 to 30, with higher scores indicating greater tendency to goal reengagement.

^j^ Subscale scores range from 0 to 21, with higher scores indicating greater anxiety and depressive symptoms.

^k^ Self-rated scores range from 0 to 40, with higher scores indicating greater impairment.

### Missing Data

Baseline characteristics between the 210 participants with complete data vs the 74 with incomplete data are presented in the eTable in Supplement 2. Moderate differences were observed in age, site, rurality, cancer stage, QOL, and depression and were used as covariates in outcome analyses.

#### Participation Satisfaction and Ability, Productivity, and Individual Activity Targets

Table 2 presents model-estimated means and between-group contrasts at each assessment time point, with baseline survey scores as covariates. PROMIS scores (including the primary measure of PROMIS satisfaction) and productivity outcomes of both groups improved over time; however, between-group trajectories were not significantly different. Measures of intervention effect for PROMIS satisfaction (week 20: Cohen *d*, 0.1 [95% CI, −0.09 to 0.29] and week 44: Cohen *d*, −0.08 [95% CI, −0.27 to 0.11]) and ability (week 20: Cohen *d*, 0.15 [95% CI, −0.06 to 0.37] and week 44: Cohen *d*, −0.08 [95% CI, −0.27 to 0.11]) were not statistically significant. The mean PROMIS score improvement from baseline to final assessment surpassed the threshold for clinically meaningful improvement in both groups (within-group change for satisfaction at week 44: BA/PS group, 5.10 [95% CI, 4.01 to 6.18] points and control group, 5.39 [95% CI, 4.29 to 6.49] points; within-group change for ability, BA/PS group, 4.08 [95%CI, 3.08 to 5.07] points; control group, 5.15 [95%CI, 4.14 to 6.16] points).

**Table 2.** Intervention Effects on the Primary Outcomes of Participation and Productivity

Outcome by week	Score, Mean (SE)		Between-group comparisons					
			Contrast		Effect size	Group by time interaction		
	BA/PS intervention	Attention control	Ratio or difference (SE)^a^	*P* value	Mean ratio or Cohen *d* (95% CI)^b^	*F* test	*P* value	
							Raw	Adjusted
Disability days past month								
0	1.32 (0.17)	1.15 (0.15)	1.14 (0.18)	.41	1.14 (0.83 to 1.57)	F_3,717.0_ = 0.7	.55	.55
8	1.03 (0.14)	0.91 (0.12)	1.14 (0.20)	.46	1.14 (0.81 to 1.61)			
20	1.22 (0.16)	0.87 (0.12)	1.4 (0.24)	.06	1.40 (0.99 to 1.97)			
44	0.88 (0.13)	0.76 (0.11)	1.17 (0.23)	.42	1.17 (0.80 to 1.71)			
WLQ^c^								
0	34.70 (1.58)	34.15 (1.60)	0.55 (2.17)	.80	0.03 (−0.20 to 0.26)	F_3,394.7_ = 0.18	.91	.99
8	27.87 (1.71)	25.35 (1.67)	2.52 (2.32)	.28	0.14 (−0.11 to 0.38)			
20	25.13 (1.73)	23.57 (1.69)	1.55 (2.34)	.51	0.08 (−0.17 to 0.33)			
44	22.79 (1.73)	21.14 (1.75)	1.65 (2.40)	.49	0.09 (−0.17 to 0.35)			
PROMIS satisfaction T score^d^								
0	45.75 (0.43)	46.00 (0.44)	−0.24 (0.60)	.68	−0.04 (−0.21 to 0.14)	F_3,747.0_ = 0.88	.45	.54
8	48.09 (0.47)	47.61 (0.46)	0.48 (0.63)	.44	0.07 (−0.11 to 0.26)			
20	50.47 (0.47)	49.81 (0.47)	0.65 (0.64)	.30	0.1 (−0.09 to 0.29)			
44	50.85 (0.48)	51.39 (0.49)	−0.54 (0.66)	.41	−0.08 (−0.27 to 0.11)			
PROMIS ability T score^e^								
0	43.35 (0.40)	43.48 (0.41)	−0.13 (0.55)	.82	−0.02 (−0.23 to 0.18)	F_3,746.4_ = 2.64	.05	.09
8	45.55 (0.43)	44.95 (0.43)	0.60 (0.58)	.30	0.11 (−0.10 to 0.33)			
20	47.27 (0.43)	46.44 (0.43)	0.82 (0.59)	.16	0.15 (−0.06 to 0.37)			
44	47.43 (0.44)	48.63 (0.45)	−1.20 (0.61)	.05	−0.22 (−0.45 to 0)			

Abbreviations: BA/PS, behavioral activation and problem-solving; PROMIS, Patient-Reported Outcomes Measurement Information System; WLQ, Work Limitations Questionnaire.

^a^ Between-group contrast is presented as ratio (SE) for disability days past month and as mean (SE) scale score point difference for WLQ and PROMIS T scores.

^b^ Between-group effect size is presented as mean ratio (95% CI) for disability days past month and as Cohen *d* (95% CI) for WLQ and PROMIS T scores.

^c^ Assessed in those who were working (n = 152); the WLQ assessed productivity with an overall score indicating the percentage decrement in productivity in the previous 2 weeks across 4 subscales (time, physical, mental or interpersonal, and output), in which scores ranged from 0% to 100%, with higher scores indicating greater decrement in productivity.

^d^ T scores range from 0 to 100, with higher scores indicating greater satisfaction in social roles and activities.

^e^ T scores range from 0 to 100, with higher scores indicating greater ability to participate in social roles and activities.

Table 3 presents the COPM individual activity target scores. Both groups reported increases in activity satisfaction and performance. The improvements were clinically meaningful^26^ and significantly greater in the BA/PS group for satisfaction (mean [SE] difference in change score, 1.25 [0.22]; *P* < .001; Cohen *d*, 0.76 [95% CI, 0.48-1.02]) and performance (mean [SE] difference in change score, 0.85 [0.20]; *P* < .001; Cohen *d*, 0.60 [95% CI, 0.32-0.87]).

**Table 3.** Intervention Effects on Primary Outcomes, Individual Activity Targets

COPM subscales^a^	Score, Mean (SE)				Between-group comparisons			
	BA/PS intervention		Attention control					
	Session 1	Session 9	Session 1	Session 9	Mean (SE) scale score points	*P* value		Cohen *d* (95% CI)
						Raw	Adjusted	
Activity importance	8.66 (0.08)	8.78 (0.09)	8.87 (0.08)	8.75 (0.09)	0.24 (0.13)	.06	.09	0.26 (−0.01 to 0.54)
Activity performance	4.13 (0.12)	6.99 (0.13)	4.29 (0.12)	6.30 (0.13)	0.85 (0.20)	<.001	<.001	0.60 (0.32 to 0.87)
Activity satisfaction	3.74 (0.14)	7.15 (0.15)	3.93 (0.14)	6.08 (0.15)	1.25 (0.22)	<.001	<.001	0.76 (0.48 to 1.02)

Abbreviations: BA/PS, behavioral activation and problem-solving; COPM, Canadian Occupational Performance Measure.

^a^ Participants rated up to 5 self-selected activities using Likert scales (ranging from 1 to 10) for 3 characteristics: importance, performance, and satisfaction. Higher scores indicated greater participation.

### QOL and Adaptive Coping, Goal Adjustment, and Distress Outcomes

Table 4 presents model-estimated outcome means and between-group contrasts at each assessment time point. Quality of life and distress outcomes improved over time for participants in both groups. The overall mean QOL improvement scores on the FACT-G from baseline to the final assessment surpassed the threshold for clinically meaningful improvement in both groups (within-group change for the BA/PS intervention group, 6.50 points; within-group change for the attention control group, 6.78 points). Participants in both groups reported increased adaptive coping over time, with a slight decrease at the final assessment and no significant between-group differences. Results indicated a significant intervention effect in goal disengagement scores. For this outcome, the baseline adjustment in the model reduced a mild baseline chance imbalance (from Cohen *d*, 0.27 to Cohen *d*, 0.10) (Table 1). On average, BA/PS participants had higher Goal Adjustment and Disengagement Scale scores than control participants at all postbaseline time points (all Cohen *d*, >0.20; all adjusted *P* < .05).

**Table 4.** Intervention Effects on Secondary and Exploratory Outcomes

Outcome by week	Score, Mean (SE)		Between-group comparisons					
			Contrast		Effect size	Group by time interaction		
	BA/PS intervention	Attention control	Difference (SE), scale scores	*P* value	Cohen *d* (95% CI) 95% CI	*F* test	*P* value	
							Raw	Adjusted
**FACT-G^a^**								
Overall								
0	69.66 (0.87)	69.45 (0.89)	0.21 (1.19)	.86	0.01 (−0.13 to 0.15)	*F*_3,731.3_ = 0.03	.99	.99
8	73.44 (0.93)	73.36 (0.92)	0.08 (1.25)	.95	0 (−0.14 to 0.15)			
20	76.25 (0.93)	76.49 (0.93)	−0.23 (1.26)	.85	−0.01 (−0.16 to 0.13)			
44	76.16 (0.95)	76.23 (0.97)	−0.07 (1.30)	.96	0 (−0.15 to 0.15)			
Physical								
0	17.21 (0.31)	17.31 (0.32)	−0.09 (0.43)	.82	−0.02 (−0.17 to 0.14)	*F*_3,744.5_ = 0.23	.87	.99
8	18.96 (0.33)	18.82 (0.33)	0.14 (0.45)	.76	0.03 (−0.14 to 0.19)			
20	19.78 (0.33)	20.01 (0.33)	−0.22 (0.45)	.62	−0.04 (−0.21 to 0.13)			
44	19.59 (0.34)	19.55 (0.35)	0.04 (0.47)	.93	0.01 (−0.16 to 0.18)			
Social								
0	19.79 (0.31)	19.64 (0.32)	0.15 (0.43)	.73	0.02 (−0.12 to 0.17)	*F*_3,740.6_ = 0.32	.81	.99
8	19.39 (0.33)	19.69 (0.33)	−0.30 (0.45)	.51	−0.05 (−0.2 to 0.1)			
20	20.14 (0.34)	20.07 (0.34)	0.07 (0.46)	.88	0.01 (−0.14 to 0.16)			
44	19.76 (0.34)	19.97 (0.35)	−0.21 (0.47)	.65	−0.04 (−0.19 to 0.12)			
Emotional								
0	15.99 (0.24)	16.01 (0.24)	−0.02 (0.33)	.95	0 (−0.14 to 0.14)	*F*_3,743.0_ = 0.46	.71	.99
8	17.2 (0.25)	16.75 (0.25)	0.45 (0.34)	.20	0.10 (−0.05 to 0.24)			
20	17.66 (0.26)	17.48 (0.25)	0.18 (0.35)	.61	0.04 (−0.11 to 0.19)			
44	17.6 (0.26)	17.55 (0.26)	0.05 (0.36)	.89	0.01 (−0.14 to 0.16)			
Functional								
0	16.56 (0.31)	16.59 (0.31)	−0.02 (0.42)	.95	0 (−0.16 to 0.15)	*F*_3,746.1_ = 0.3	.83	.99
8	17.78 (0.33)	18.17 (0.33)	−0.39 (0.44)	.38	−0.07 (−0.24 to 0.09)			
20	18.58 (0.33)	19.01 (0.33)	−0.43 (0.45)	.33	−0.08 (−0.25 to 0.08)			
44	19.11 (0.33)	19.28 (0.34)	−0.16 (0.46)	.72	−0.03 (−0.2 to 0.14)			
**Brief-COPE^b^**								
Active								
0	6.35 (0.11)	6.48 (0.11)	−0.13 (0.15)	0.36	−0.09 (−0.27 to 0.1)	*F*_3,746_ = 0.8	.50	.99
8	6.68 (0.11)	6.58 (0.11)	0.10 (0.16)	0.50	0.07 (−0.13 to 0.26)			
20	6.68 (0.12)	6.58 (0.12)	0.10 (0.16)	0.52	0.07 (−0.13 to 0.26)			
44	6.55 (0.12)	6.35 (0.12)	0.19 (0.16)	0.24	0.12 (−0.08 to 0.33)			
Planning								
0	6.33 (0.12)	6.54 (0.12)	−0.21 (0.16)	0.19	−0.13 (−0.32 to 0.06)	*F*_3,744.8_ = 2.93	.03	.21
8	6.80 (0.13)	6.58 (0.12)	0.22 (0.17)	0.20	0.13(−0.07 to 0.33)			
20	6.95 (0.13)	6.53 (0.13)	0.42 (0.17)	0.02	0.25 (0.05 to 0.46)			
44	6.52 (0.13)	6.50 (0.13)	0.02 (0.18)	0.91	0.01 (−0.2 to 0.22)			
Reframing								
0	6.35 (0.11)	6.48 (0.11)	−0.14 (0.15)	0.37	−0.08 (−0.26 to 0.1)	*F*_3,742_ = 0.21	.89	.99
8	6.60 (0.12)	6.57 (0.12)	0.03 (0.16)	0.85	0.02 (−0.17 to 0.21)			
20	6.58 (0.12)	6.56 (0.12)	0.01 (0.16)	0.92	0.01 (−0.18 to 0.2)			
44	6.40 (0.12)	6.48 (0.12)	−0.08 (0.17)	0.62	−0.05 (−0.24 to 0.15)			
**GDGRS**								
Disengagement^c^								
0	10.3 (0.21)	10.6 (0.22)	0.10 (0.30)	0.30	−0.10 (−0.28 to 0.09)	*F*_3,741.2_ = 5.09	.002	.02
8	11.3 (0.23)	10.67 (0.22)	0.12 (0.32)	0.04	0.20 (0.01 to 0.40)			
20	11.29 (0.23)	10.43 (0.23)	−0.05 (0.32)	0.01	0.28 (0.08 to 0.47)			
44	11.47 (0.23)	10.39 (0.24)	0.22 (0.33)	0	0.35 (0.14 to 0.55)			
Reengagement^d^								
0	21.26 (0.27)	21.14 (0.28)	−0.15 (0.28)	0.74	0.03 (−0.16 to 0.22)	*F*_3,745.7_ = 0.81	.49	.99
8	21.40 (0.29)	21.81 (0.29)	−0.16 (0.30)	0.30	−0.11 (−0.31 to 0.1)			
20	22.03 (0.30)	22.01 (0.29)	−0.31 (0.30)	0.97	0 (−0.2 to 0.21)			
44	21.50 (0.30)	22.03 (0.31)	0.31 (0.31)	0.20	−0.14 (−0.35 to 0.07)			
**HADS^e^**								
Anxiety								
0	9.08 (0.22)	8.98 (0.23)	0.10 (0.30)	0.75	0.02 (−0.11 to 0.16)	*F*_3,737.5_ = 0.17	.91	.99
8	7.79 (0.24)	7.68 (0.24)	0.12 (0.32)	0.71	0.03 (−0.12 to 0.17)			
20	7.44 (0.24)	7.49 (0.24)	−0.05 (0.32)	0.87	−0.01 (−0.16 to 0.13)			
44	7.42 (0.24)	7.19 (0.25)	0.22 (0.33)	0.50	0.05 (−0.10 to 0.20)			
Depression								
0	6.13 (0.21)	6.28 (0.21)	−0.15 (0.28)	0.60	−0.04 (−0.19 to 0.11)	*F*_3,733.6_ = 0.85	.47	.99
8	5.17 (0.22)	5.33 (0.22)	−0.16 (0.30)	0.60	−0.04 (−0.20 to 0.11)			
20	4.87 (0.22)	5.18 (0.22)	−0.31 (0.30)	0.31	−0.08 (−0.24 to 0.08)			
44	5.2 (0.23)	4.89 (0.23)	0.31 (0.31)	0.32	0.08 (−0.08 to 0.24)			

Abbreviations: BA/PS, behavioral activation and problem-solving; FACT-G, Functional Assessment of Cancer Therapy-General; GDGRS, Goal Disengagement and Goal Reengagement Scale; HADS, Hospital Anxiety and Depression Scale.

^a^ A 27-item questionnaire with 4 subscales: physical, social, emotional, and functional well-being. A 5-point increase in overall score indicates meaningful improvement. Scores range from 0 to 108, with higher scores indicating greater quality of life.

^b^ Scores range from 0 to 6, with higher scores indicating greater use of the coping strategy.

^c^ Scores range from 4 to 20, with higher scores indicating greater tendency to goal disengagement.

^d^ Scores range from 6 to 30, with higher scores indicating greater tendency to goal reengagement.

^e^ Subscale scores range from 0 to 21, with higher scores indicating greater anxiety and depressive symptoms.

## Discussion

In this RCT, we enrolled and retained the target sample despite the backdrop of the COVID-19 pandemic.^24^ The sample underrepresented Hispanic women (2.1% of the sample); however, the proportion of Black women (16.5% of the sample) mirrored the proportion of US Black women (14.0%).^48^ The sample was generally well-educated, and two-thirds of the sample had at least a bachelor’s degree, which is greater than the 38% of the US population reporting a college degree in the 2021 US Census.^49^

Contrary to our hypotheses, the BA/PS condition outperformed the attention control condition only for individual activity targets and goal disengagement. The individual activity targets reflected participants’ priorities for functional recovery; it is encouraging that the BA/PS tailored focus on adapting activities and goals optimized individuals’ ability to perform valued activities. This finding resonates with our process evaluation,^24^ in which BA/PS participants reported significantly greater benefits than control participants regarding adjusting habits and routines, setting goals, and getting exercise.

Participants in the BA/PS group reported increased goal disengagement (ie, one’s tendency to withdraw effort from untenable goals). This is logical because the last step of the BA/PS intervention assessed the degree of goal attainment and satisfaction with executing a weekly action plan. The attention control condition had no such process. Goal disengagement can have a protective effect on well-being.^50,51^ Psychooncology^52^ and rehabilitation^53^ studies have also reported increased goal disengagement, suggesting that goal-setting interventions may support realistic goal appraisal leading to better use of individuals’ time and energy. Higher goal disengagement scores are most effective when combined with goal reengagement,^54^ as occurred in our results.

In the absence of a no-treatment condition, we were not able to determine whether the clinically meaningful improvements in both groups’ participation and QOL scores were due to time alone. However, there are 3 reasons that we do not think natural recovery offers the best explanation of our results. First, other studies reported decreased social participation and life satisfaction^55^ and physical activity^9,56^ during the COVID-19 pandemic. Our sample’s improvement on these variables runs counter to the decline that the pandemic restrictions seemed to produce. Second, our group’s pilot study demonstrated no improvement in functional well-being during a 6-week, no-treatment run-in phase.^14^ Finally, close to half of our sample enrolled more than 6 months since completing treatment. The passage of time had not yet reduced their restrictions, which suggests that natural recovery may not adequately explain our results.

Alternatively, our process evaluation indicated that the educational content about self-management in the attention control condition may have been more potent than anticipated.^24^ Similar psychoeducation programs have improved QOL^57^ and emotional well-being,^58,59^ with attention from nurses reported as highly valued by participants.^60^ Attention may have been particularly potent and appreciated during the pandemic when many participants were experiencing isolation from social distancing mandates.^24^ In sum, there may be multiple paths to reducing participation restrictions. The BA/PS condition may reduce restrictions by repeated practice of self-selected valued activities; the attention control condition might reduce restrictions by providing education and support to manage late effects of cancer treatment.

### Limitations

A limitation of this study is that oral administration of measures may have introduced some social desirability if participants minimized their limitations, although it would not have differentially affected the 2 conditions. Future research should consider using objective measures (eg, actigraphy to quantify movement, geolocation to quantify time spent outside the home) or real-time measures (eg, ecological momentary assessment) that may reduce bias or be more sensitive to changes from BA/PS participants’ execution of individualized goals and action plans. The coaches administering the self-rated COPM were not blind to conditions; however, participants were not prompted to record and were not reminded of their baseline COPM ratings during readministration 20 weeks later. Finally, we were unable to verify diagnosis, clinical characteristics, and comorbidities of women recruited via Social media, as we did not have access to their medical records.

## Conclusions

In this multisite RCT of a telephone-based coaching intervention, which achieved target enrollment and high intervention fidelity, the BA/PS intervention catalyzed greater improvements in individual activity targets and higher goal disengagement but did not outperform an education-based attention control condition on other outcomes. Future research is needed to determine whether time alone or both conditions’ focus on individualized support and elicitation of recovery priorities led to improvements or whether objective measures may illuminate the potential of BA/PS to foster cancer survivors’ functional recovery.

## Notes

Supplement 1.
Trial Protocol

Supplement 2. **eTable.** Association Between Participant Characteristics and Missing Data at Follow-Up

Supplement 3.
Data Sharing Statement
