# Error in Abstract

**DOI:** 10.1001/jamanetworkopen.2025.1740

**Published:** 2025-02-20

In the Original Investigation titled “Telephone-Based Rehabilitation Intervention to Optimize Activity Participation After Breast Cancer: A Randomized Clinical Trial,”^1^ published March 22, 2024, the total number of patients in the intervention group was incorrectly stated as 114 in the abstract; the correct number is 144. This article has been corrected.
