# Supplementary material for: Telephone-Based Rehabilitation Intervention to Optimize Activity Participation After Breast Cancer: A Randomized Clinical Trial
Source: JAMA Netw Open. 2024 Mar 22;7(3):e242478. doi: 10.1001/jamanetworkopen.2024.2478 (PMC10960198; doi:10.1001/jamanetworkopen.2024.2478)
Supplement: Supplement 1. — Trial Protocol [file jamanetwopen-e242478-s001.pdf]

A PHASE III, RANDOMIZED, SINGLE BLIND, ATTENTION CONTROLLED, MULTI-CENTER STUDY OF THE EFFECTS OF A REHABILITATION INTERVENTION ON PARTICIPATION RESTRICTIONS OF FEMALE BREAST CANCER SURVIVORS

Regulatory Sponsor: *Kathleen Lyons, ScD, OTR/L*
Psychiatry Department
HB 7750, DHMC
603-653-3656

Funding Sponsor: *Pending Council Review at the National Cancer Institute*

Study Product: *Behavioral Activation/Problem-solving (BA/PS) Rehabilitation Intervention*

Protocol Number: D18167

IND Number: *N/A*

Initial version: 7/18/18
Amended: [date]
Amended: [date]

Table of Contents

STUDY SUMMARY **1**
1 INTRODUCTION **3**
1.1 BACKGROUND 3
1.2 INVESTIGATIONAL AGENT 4
1.3 PRECLINICAL DATA 4
1.4 CLINICAL DATA TO DATE 4
1.5 DOSE RATIONALE AND RISK/BENEFITS 6
2 STUDY OBJECTIVES **6**
3 STUDY DESIGN **6**
3.1 GENERAL DESIGN ..... **ERROR! BOOKMARK NOT DEFINED.**
3.2 PRIMARY STUDY ENDPOINTS 7
3.3 SECONDARY STUDY ENDPOINTS 7
3.4 PRIMARY SAFETY ENDPOINTS 7
4 SUBJECT SELECTION AND WITHDRAWAL **7**
4.1 INCLUSION CRITERIA 7
4.2 EXCLUSION CRITERIA 8
4.3 SUBJECT RECRUITMENT AND SCREENING 8
4.4 EARLY WITHDRAWAL OF SUBJECTS 9
4.4.1 When and How to Withdraw Subjects 9
4.4.2 Data Collection and Follow-up for Withdrawn Subjects 9
5 STUDY DRUG **9**
5.1 DESCRIPTION 9
5.2 TREATMENT REGIMEN 9
5.3 METHOD FOR ASSIGNING SUBJECTS TO TREATMENT GROUPS 11
5.4 PREPARATION AND ADMINISTRATION OF STUDY DRUG 11
5.5 SUBJECT COMPLIANCE MONITORING 11
5.6 PRIOR AND CONCOMITANT THERAPY 11
5.7 PACKAGING 11
5.8 BLINDING OF STUDY DRUG 11
5.9 RECEIVING, STORAGE, DISPENSING AND RETURN 11
5.9.1 Receipt of Drug Supplies 11
5.9.2 Storage 11
5.9.3 Dispensing of Study Drug 11
5.9.4 Return or Destruction of Study Drug 12
6 STUDY PROCEDURES **12**
6.1 VISIT 1 ..... **ERROR! BOOKMARK NOT DEFINED.**
6.2 VISIT 2 ..... **ERROR! BOOKMARK NOT DEFINED.**
6.3 ETC. ..... **ERROR! BOOKMARK NOT DEFINED.**
7 STATISTICAL PLAN **13**
7.1 SAMPLE SIZE DETERMINATION 13
7.2 STATISTICAL METHODS 14
7.3 SUBJECT POPULATION(S) FOR ANALYSIS 16
8 SAFETY AND ADVERSE EVENTS **16**
8.1 DEFINITIONS 16
8.2 RECORDING OF ADVERSE EVENTS 17

8.3 Reporting of Serious Adverse Events and Unanticipated Problems 18
8.3.1 Investigator reporting: notifying the study sponsor 18
8.3.2 Investigator reporting: notifying the Dartmouth IRB 18
8.3.3 Investigator reporting: Notifying a non-Dartmouth IRB 19
8.3.4 Sponsor reporting: Notifying the FDA 19
8.3.5 Sponsor reporting: Notifying participating investigators 19
8.4 Unblinding Procedures 19
8.5 Stopping Rules 19
8.6 Medical Monitoring 19
8.6.1 Internal Data and Safety Monitoring Board 20
8.6.2 Independent Data and Safety Monitoring Board 20

9 DATA HANDLING AND RECORD KEEPING 20

9.1 Confidentiality 20
9.2 Source Documents 20
9.3 Case Report Forms 21
9.4 Records Retention 21

10 STUDY MONITORING, AUDITING, AND INSPECTING 21

10.1 Study Monitoring Plan 21
10.2 Auditing and Inspecting 21

11 ETHICAL CONSIDERATIONS 21

12 STUDY FINANCES 22

12.1 Funding Source 22
12.2 Conflict of Interest 22
12.3 Subject Stipends or Payments 22

13 PUBLICATION PLAN 22

14 REFERENCES 22

15 ATTACHMENTS 22

List of Abbreviations

AE: Adverse event
BA/PS: Behavioral Activation/Problem-solving
DSMC: Data and Safety Monitoring Committee
DHMC: Dartmouth-Hitchcock Medical Center
DSM: Data Safety Monitoring
DSMB: Data Safety Monitoring Board
EC: Ethics Committee
EC/IRB: Ethics Committee/Internal Review Board
CART: Classification and Regression Trees
CFR: Case report form
FACT-B: Functional Assessment of Cancer Therapy-Breast Cancer
FACT-G: Functional Assessment of Cancer Therapy-General
FDA: Federal Drug Administration
FDR: False Discovery Rate
GDGRS: Goal Disengagement and Goal Reengagement Scale
HIPAA: Health Insurance Portability and Accountability Act
IRB: Internal Review Board
MAR: Missing at Random
MCAR: Missing Completely at Random
MEPS: Medical Expenditure Panel Survey
MNAR: Not Missing at Random
MID: Minimally important differences
N: Population size
N/D: not defined
N/A: not applicable
NCCC: Norris Cotton Cancer Center
PET: Positron emission tomography scan
PHI: Protected Health Information
PI: Principal Investigator
PROMIS: Patient-reported Outcomes Measurement Information System
R: is a programming language and free software environment for statistical computing and graphics
RCT: Randomized Control Trial
SAS: statistical analysis software
UAB or UABCCC: University of Alabama at Birmingham Comprehensive Cancer Center
UPR: Unanticipated Problem Involving Risks to Subjects or Others
US: United States
WSAS: Work and Social Adjustment Scale
WLQ: Work Limitations Questionnaire

Study Summary

Title	***A PHASE III, RANDOMIZED, SINGLE BLIND, ATTENTION CONTROLLED, MULTI-CENTER STUDY OF THE EFFECTS OF A REHABILITATION INTERVENTION ON PARTICIPATION RESTRICTIONS OF FEMALE BREAST CANCER SURVIVORS***
Short Title	RCT of Behavioral Activation/Problem-solving Rehabilitation Intervention for Breast Cancer Survivors
Protocol Number	D18167
Phase	Phase 3
Methodology	Single blind; Randomized, attention control
Study Duration	3.5 years
Study Center(s)	Multi-center (DHMC and University of Alabama at Birmingham Comprehensive Cancer Center).
Objectives	**Aim 1:** To test the effect of BA/PS on *participation* in roles and activities of breast cancer survivors.<br>**Hypothesis 1a (primary: participation satisfaction):** Compared to attention control participants, BA/PS participants will report greater participation as measured by the Patient-reported Outcomes Measurement Information System (PROMIS) Satisfaction with Social Roles and Activities Short Form 8a.<br>**Hypothesis 1b (secondary: participation ability and productivity):** Compared to attention control participants, BA/PS participants will report higher activity performance as measured by the PROMIS Ability to Participate in Social Roles and Activities Short Form 8a and higher productivity as measured by the Disability Days Section of the Medical Expenditure Panel Survey and the Work Limitations Questionnaire.<br>**Aim 2:** To test the effect of BA/PS on *quality of life* of breast cancer survivors.<br>**Hypothesis:** Compared to attention control participants, BA/PS participants will report higher quality of life as measured by the Functional Assessment of Cancer Therapy- Breast Cancer (FACT-B).<br>**Exploratory Aim:** To test the effect of BA/PS on the outcomes of *coping*, *goal adjustment*, and *distress*.<br>**Hypothesis:** Compared to attention control participants, BA/PS participants will report greater adaptive coping (Brief COPE), greater goal adjustment (Goal Disengagement and Goal Reengagement Scale), and less distress (PROMIS Emotional Distress- Depression- Short Form 8a).
Number of Subjects	300
Diagnosis and Main Inclusion Criteria	Females diagnosed with Stage 1-3 breast cancer and within one year of completion of locoregional treatment and/or adjuvant therapy with curative intent and absence of disease recurrence.
Study Product, Dose, Route, Regimen	Behavioral Activation/Problem-solving (BA/PS) Rehabilitation Intervention
Duration of administration	9 sessions
Reference therapy	Attention Control

Statistical Methodology	A longitudinal model fitted with linear mixed methods will be used compare the two groups' functional recovery over time according to each outcome.

1 Introduction

This document is a protocol for a human research study. This study is to be conducted according to US and international standards of Good Clinical Practice (FDA Title 21 part 312 and International Conference on Harmonization guidelines), applicable government regulations and Institutional research policies and procedures.

1.1 Background

Definition of participation. This proposal is focused on breast cancer survivors' ability to engage in life, fulfill social roles, and perform activities and daily routines. Disability scholars use various labels for this construct**.** We are using the language of the **World Health Organization**, where the word *"**participation**"* describes a *state of health and functioning in which a person can fully engage in roles and life situations*.[1]

Operationalization. The construct of participation is multidimensional.[2] The objective aspects of participation include whether and how often an activity is performed; the subjective aspects include the level of difficulty and satisfaction with activity engagement.[3] Most measures of participation focus on activities that fulfill social roles within particular environments, according to personal, societal, and cultural standards.[2] As such, measures of participation generally privilege the individual's perspective as he or she has the best vantage point from which to judge the degree to which he or she is fulfilling various roles within home and community settings.

Prevalence. A population-based study (in which the most common diagnosis for women was breast cancer) reported 31% of both recent and long-term cancer survivors reported restrictions in their ability to participate in roles and life activities. This proportion was significantly more than the 13% of age-matched controls without cancer reporting participation restrictions.[4] In a 2017 study of 245 breast cancer survivors, 90% reported at least some difficulty completing work activities, 87% reported difficulty doing strenuous activities, 78% reported difficulty doing moderate activities, and 74% reported difficulty completing household activities.[5]

Persistence. There is no compelling evidence to suggest that participation restrictions resolve naturally over time. While the studies cited above did not use a longitudinal design, the proportions of recent and long-term survivors reporting participation restrictions are remarkably similar.[4] Research indicates that even after underlying physical impairments have resolved, limitations in recreational activities, sexual activities, work, and daily activities can persist two[6] to six[7] years after breast cancer treatment.

Causes and consequences. Multiple factors interact to restrict activity participation, and co-morbidities play a large role in generating disability for cancer survivors.[8,9] Regardless of the etiology, participation restrictions affect both quantity and quality of life. Lower participation in valued activities predicts shorter overall survival in women treated for breast cancer.[10-12] In samples of women living with or recovering from breast cancer, self-reported disruption in daily activities is associated with greater depression[13] and is predictive of less positive mood over time.[14] As such, optimizing participation and promoting functional recovery are important aspects of cancer survivorship.[15,16]

Scientific Premise

Gap. We lack evidence-based interventions that directly target participation in roles and life activities.[17] Self-management interventions and multidimensional survivorship programs improve symptom management, distress, and quality of life, yet they do not consistently or conclusively improve the outcomes of social functioning or role functioning that are most similar to participation.[18,19] Rehabilitation interventions primarily address physical impairments. While important, impairment reduction alone may not improve activity participation. For example, a recent meta-analysis[20] found that exercise improves social and emotional well-being of breast cancer survivors, but does not have a significant effect on functional well-being (the aspect of quality of life that is most similar to the construct of participation[21]). Further, because not all underlying impairments can be remediated, adaptive approaches directly targeting activity participation are needed.

Premise. Cheville and colleagues found that the number of physical impairments explained only half of the variance in participation restrictions reported by women with advanced breast cancer.[22] Recent models of cancer rehabilitation acknowledge that participation in roles and activities is influenced not only by physical impairments, but also by other personal factors, environmental factors, and activity demands.[23] We assert that a structured intervention can increase breast cancer survivors' active coping and proficiency in manipulating the environment and adapting activities and that weekly application of those skills can lead to enhanced activity participation. Support for this assertion is presented in Section C.2., where we describe our preliminary studies. Our approach is designed to catalyze functional recovery by encouraging women to take strategic, incremental actions to optimize activity engagement, without waiting for symptoms and side effects to fully resolve.

Changing the Field. This BA/PS intervention could be utilized by rehabilitation therapists, nurses, and social workers in clinical or workplace environments. Occupational therapists, in particular, provide billable services with the ultimate goal of maximizing the ability to function at home and in the community.[24] The BA/PS intervention provides a standardized way to move beyond treating impairments and to directly optimize the ability to engage in activities related to valued roles.

1.2 Investigational Agent

Not applicable.

1.3 Preclinical Data

Not applicable.

1.4 Clinical Data to Date

Preliminary Studies Supporting the Scientific Premise

Overview. Our team has conducted pilot studies to establish the feasibility of our methods and demonstrate the intervention's acceptability and potential efficacy. We describe below how we addressed issues that allow us to feel confident that the proposed RCT will be successful.

Acceptability of randomization. In our first pilot study, 31 women undergoing chemotherapy for breast cancer were randomized to the intervention or usual care.[25] We learned that our participants would accept randomization and those assigned to usual care had adequate retention in the 3-month study (94%). Additionally, in our team's larger studies of similar interventions (two RCTs of a supportive care intervention each with >250 participants[26,27] and an RCT of a home-based BA/PS intervention for 61 older adults with cancer[28]) we have successfully randomized participants and kept data collectors blind to group assignment.

Potential efficacy. After completing the first pilot RCT,[25] we enrolled 32 women who had completed breast cancer treatment and were experiencing participation restrictions (per the screening tool that will be used in this study) into two studies, each using a single arm study design.[29] One of the studies included a no-treatment run-in phase to assess the stability of functional limitations after cancer treatment. There was no change in quality of life during the 6-week no-treatment run-in phase. A longitudinal analysis showed a main effect of time for overall quality of life (as measured by the Functional Assessment of Cancer Therapy-Breast; FACT-B; $F(5, 43.1) = 5.1$, $p = 0.001$), including a significant improvement in functional well-being. Women reported an average increase of 10 points on the FACT quality of life measure immediately after the intervention. A change in 5 points has been found to signal clinically meaningful improvement on the FACT total score.[30,31] There were also significant improvements in adaptive coping (as measured by the Brief COPE), namely active coping ($F(3, 31.7) = 4.9$, $p = 0.007$), planning ($F(3, 36.0) = 4.1$, $p = 0.01$), and reframing ($F(3, 29.3) = 8.5$, $p < 0.001$).[29]

Use of BA/PS to address various activities. A content analysis of session data from the first pilot study indicated that participants did not rely exclusively on one type of adaptive strategy, but brainstormed diverse strategies that changed *what* activities were done (32% of solutions), and *where* (10%), *when* (21%), with *whom* (16%), and *how* (21%) they were done.[32] The content analysis also revealed that

women used the intervention to address 11 types of challenging activities (e.g., exercise, instrumental activities of daily living, work, socializing). In the subsequent two studies using the BA/PS structure, we similarly demonstrated that one parsimonious structure could address up to 13 different types of activities[28,33] according to the participant priorities. When analyzing the types of goals set by participants in those two studies, we demonstrated that BA/PS targets both the ability to perform activities as well as satisfaction with performance.[33]

Feasibility of telephone delivery. We delivered the intervention by telephone in our three pilot studies involving women with breast cancer[25,29] because of feedback from participants in our previous studies[26,34,35] who consistently appreciated that our interventions do not require them to return to or extend their stay at the cancer center. In our Alabama site, Dr. Bakitas has been able to recruit participants to similar studies of individually-tailored, telephone-delivered interventions and has found that having a local recruiter and interventionist (e.g., with a local accent) has allowed for successful recruitment and retention.

Summary. Our pilot studies used a structured, problem-solving and action planning approach to find ways to increase participation in valued daily activities. We have used participant feedback to create a standardized treatment manual that flexibly addresses the individual needs of cancer survivors. Definitive efficacy testing is warranted as the studies demonstrate the feasibility, acceptability, and potential efficacy of our approach.

Case Example of BA/PS to Illustrate the Significance and Scientific Premise

"Amy" was a 53-year old woman enrolled in our third pilot study. She had a mastectomy to treat her Stage II breast cancer followed by 18 weeks of chemotherapy and 8 weeks of radiation. She was seen by physical therapy to address her severe fatigue, peripheral neuropathy, and lymphedema in her right arm.

Amy reported three challenges with daily activities. First, she was working 24 hours a week, but felt exhausted and overwhelmed at work. Her long-term goal was to resume full-time work, but was currently calling in sick and coming in late and was "burning out the good will" of her employer. Second, she had lived in a large home since divorcing and now felt it was too much for her to take care of properly. She wanted to get back to regular cleaning and start to downsize her possessions. Third, because her physical challenges absorbed her coping resources, she was finding it difficult to reengage with her previous practice of meditation.

In the first BA/PS session, Amy's goal was to re-establish her daily meditation practice. She knew how to meditate, so the session focused on identifying an achievable goal and constructing an action plan that would be manageable, given the barriers of fatigue and time management she had identified. In the second session, she was happy to report that she had meditated six out of the seven days and had enjoyed the experience. In subsequent sessions, she updated her action plan to accommodate any new barriers to meditating.

In the second session, she set a goal to downsize some items in the attic. Using the BA/PS framework, she set an achievable goal and identified modifications that would help her make enough progress to feel successful, but not become exhausted. For example, she brainstormed bringing up a lawn chair to avoid sitting on the floor, setting the alarm on her phone to encourage rest periods, gathering packing supplies on the day before starting the project, and stretching her arm that morning to help her be limber. She also identified where in the attic she would start (i.e., objects that were not too heavy, not likely to stir up unhappy memories, etc.).

In the third session, Amy said she met her goals. However, she was distraught because she was meeting with her boss at the end of the week due to poor work attendance and performance. She used the BA/PS framework to explore the work challenges and brainstorm options for how to improve performance in targeted areas (e.g., organize her work files over the weekend and gather her work supplies and lunch the night before). As part of her action plan, she figured out how she would share this analysis and action plan with her boss.

In the fourth session, Amy said she shared her plan with her boss who was grateful that she had come to the meeting with a plan instead of simply an intention to do better. As a result of the meeting, she was not put on probation and she used her identified solutions to function better at work. Over the remaining sessions, she continued to set achievable goals for functioning better at work, organizing her home, and

meditating. She said that planning out manageable steps to reach her long-term goals had been invaluable and had "saved her job."

1.5 Dose Rationale and Risk/Benefits

Dose rationale. The therapies that BA/PS is built upon typically show that six sessions allow sufficient exposure for participants to meet many of their goals and independently utilize the framework.[36,37] The theoretical rationale for the three monthly sessions comes from the Transtheoretical Model[38] that suggests there is a maintenance stage of intentional behavior change where people need to actively solidify habits and develop confidence in their ability to sustain gains in activity engagement. As such, the three monthly follow-ups are booster sessions to enhance motivation, provide feedback, and keep the focus on continued incremental gains.

Risk. There are three potential risks involved in this study: (1) the risk of hurting oneself when trying to increase activity level (e.g., falling while exercising or performing home management tasks); (2) the risk of distress while talking about challenges with functional recovery after cancer treatment; and (3) risk of loss of confidentiality. The level of risk is generally quite low and strategies to minimize risks are incorporated into the BA/PS treatment manual.

Potential benefit. Participants who are randomized to the BA/PS intervention arm will receive elements of a problem-solving intervention that has been shown to improve function and quality of life in other populations. Therefore, participants may benefit from their participation in the study. If shown to be efficacious, the intervention model could be used to improve health outcomes for cancer patients across the country.

2 Study Objectives

Aim 1: To test the effect of BA/PS on *participation* in roles and activities of breast cancer survivors.

Hypothesis 1a (primary: participation satisfaction): Compared to attention control participants, BA/PS participants will report greater participation as measured by the Patient-reported Outcomes Measurement Information System (PROMIS) Satisfaction with Social Roles and Activities Short Form 8a.[39,40]

Hypothesis 1b (secondary: participation ability and productivity): Compared to attention control participants, BA/PS participants will report higher activity performance as measured by the PROMIS Ability to Participate in Social Roles and Activities Short Form 8a[39,40] and higher productivity as measured by the Disability Days Section of the Medical Expenditure Panel Survey[41] and the Work Limitations Questionnaire.[42]

Aim 2: To test the effect of BA/PS on *quality of life* of breast cancer survivors.

Hypothesis: Compared to attention control participants, BA/PS participants will report higher quality of life as measured by the Functional Assessment of Cancer Therapy- Breast Cancer (FACT-B).[43,44]

Exploratory Aim: To test the effect of BA/PS on the outcomes of *coping*, *goal adjustment*, and *distress*.

Hypothesis: Compared to attention control participants, BA/PS participants will report greater adaptive coping (Brief COPE[45]), greater goal adjustment (Goal Disengagement and Goal Reengagement Scale[46]), and less distress (PROMIS Emotional Distress- Depression- Short Form 8a.[47]).

3 Study Design

Research question. This RCT was designed to answer the question "Is BA/PS efficacious in enhancing activity participation and quality of life of breast cancer survivors?" The primary aims are to assess the efficacy of BA/PS over time (Aims 1 and 2). We will also explore other potential effects of BA/PS on adaptive coping, goal adjustment, and distress (Exploratory Aim).

Figure 1. Study design

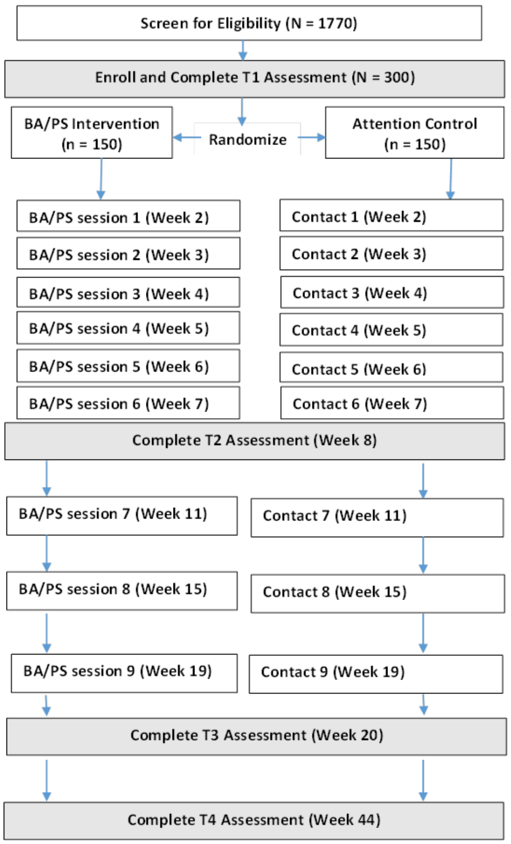

Design Overview. We will recruit 300 women over the age of 18 reporting participation restrictions after completing curative treatment for Stage 1-3 breast cancer within the past year. Half of the participants will be randomized to the 4-month BA/PS intervention which consists of 6 weekly telephone calls followed by 3 monthly telephone calls. BA/PS is designed to teach problem-solving and action planning to promote functional recovery. The other half of participants will be assigned to an attention control condition providing education about survivorship topics. This control condition will allow us to account for the effect of time and history, and the non-specific effects of attention.

Assessments. Assessments will be administered via telephone by a research assistant blind to group assignment. Participants will complete outcome assessments upon enrollment (T1) and at 8 weeks (T2), 20 weeks (T3) and 44 weeks (T4) later. The T2 assessment captures the short-term outcomes of the most intensive part of the intervention (i.e., after six weekly sessions). The T3 assessment will capture the short-term outcomes at the end of the full intervention. The T4 assessment explores the sustained effect of BA/PS (six months after BA/PS ends). Our decision to include the T4 assessment was influenced by a systematic review that indicated it often takes six months to see maximum effects of interventions targeting participation in adults with physical disabilities.[48] The study aim is to test whether the BA/PS intervention affects the "slope" of functional recovery over time. With the longitudinal data, we will also be able to explore the pace of improvement and whether the two groups differ at these clinically relevant time points.

3.1 *Primary Study Endpoints*

Efficacy regarding primary outcome of participation (satisfaction, ability and productivity).

3.2 *Secondary Study Endpoints*

Efficacy regarding secondary outcome of quality of life and exploratory outcomes of coping, goal adjustment, and distress.

3.3 *Primary Safety Endpoints*

We will measure study-related adverse events (e.g., fall during activity) to evaluate safety.

4 Subject Selection and Withdrawal

4.1 *Inclusion Criteria (with justification)*

1. Age of 18 years or older.
2. Experiencing reduced participation (i.e., a score of > 10 on the Work and Social Adjustment Scale[49]).
3. Females diagnosed with Stage 1-3 breast cancer and within one year of completion of locoregional treatment and/or adjuvant therapy with curative intent and absence of disease recurrence.

Biological variables of age and gender. In our pilot studies, the intervention has been flexible enough to be used with various challenges that occur across the age range.[25,29] This proposal's focus upon female breast cancer survivors reflects our pilot research population and allows us to focus recruitment resources with a smaller, closely aligned number of providers. We have decided to focus on females with breast

cancer because of the very low prevalence rate of breast cancer in males. Because our sites see a mean of 3.8 males each year with breast cancer, we will not enroll enough males to allow us to draw sound conclusions that are generalizable to a male population.

Medical co-morbidities. We considered the advantages and disadvantages of excluding women who have medical co-morbidities that affect their daily activities. Cancer survivors report an average of five co-morbidities[50] that interact with cancer treatment and affect activities. The most prevalent co-morbidities are hypertension, eye or ear problems, and arthritis.[50] Excluding people with such co-morbidities is conceptually appealing in that it would allow a focus on only “cancer-related disability.” However, in practice it is difficult to determine whether disability is caused by cancer, a comorbidity, or an interaction of the two. Further, comprehensive cancer rehabilitation must address both pre-existing and treatment-related conditions.[51] Because we ultimately want to develop a generalizable intervention that has broad applicability for cancer survivors experiencing disability (i.e., strong external validity), it is necessary to develop and test interventions that can address any type of participation restriction, regardless of its source. Therefore, we will not exclude potential participants solely because of medical co-morbidities.

Time since treatment. We are targeting survivors who are within one year of completing curative therapy because we are interested in supporting the middle phase of survivorship (i.e., the transition from active treatment toward extended survival).[52] We recognize that interest in and readiness for the intervention can come at different times for survivors. In our pilot studies, semi-structured interviews revealed that some women would have preferred to begin the intervention immediately after treatment ended (i.e., when they were experiencing the most difficulty re-establishing routines). Other women felt the need for intervention after 6 months had gone by (i.e., when they had a sense of their residual participation restrictions). While a one-year window of time may add heterogeneity, we think it will enhance the generalizability of the findings if BA/PS can address varying needs over time.

4.2 Exclusion Criteria

1. Non-English speaking.
2. Non-correctable hearing loss.
3. Moderate-severe cognitive impairment indicated by a score < 3 on a 6-item cognitive screener.[53]
4. History of severe mental illness (i.e., schizophrenia, bipolar disorder), current major depressive disorder, active suicidal ideation, or active substance misuse documented within the medical record.

Rationale. While the intervention is amenable to translation to other languages and modification for use with a hearing-impaired population, these adaptations are beyond the scope of the current proposal. Similarly, the highly structured program has been able to accommodate subtle cognitive difficulties[54] that are often reported after cancer treatment, but we need to exclude survivors with gross cognitive deficits that would impede safe and independent application of the action plan. Also, the needs of women with schizophrenia, bipolar disorder, major depressive disorder, suicidal ideation, and substance misuse disorders are greater than can be adequately provided by our telephone-delivered intervention. People who are excluded according to these criteria will be referred to clinic-based rehabilitation or behavioral services.

4.3 Subject Recruitment and Screening

Strategies to minimize bias in sampling. Our eligibility criteria are clearly defined so that the sample is not biased by relying upon a clinician’s determination of a given patient’s “appropriateness” or need for the study. Our use of the telephone for study and intervention procedures, along with our flexible staffing (i.e., staff available during early evening hours), allows us to reduce barriers to participation that may be experienced by working women or mothers with small children.

Procedure. Our research team has established a successful mechanism in which we collaborate with clinicians to identify eligible patients each week. We apply for a Health Insurance Portability and Accountability Act (HIPAA) waiver to allow research staff to screen clinic schedules to identify potential study participants. The research assistant consults with the clinicians to confirm eligibility based upon clinical characteristics. Clinicians deliver the brochure describing the study to patients. Our research assistant is available in the clinic or by telephone to further explain the study to patients, screen for

eligibility, and initiate informed consent procedures. We will follow the same procedures at both sites. In Alabama, the work will be conducted by the Recruitment and Retention Shared Facility of the UAB School of Medicine. Dr. Bakitas is successfully using the Shared Facility to recruit the sample for her current RCT of a palliative care intervention.

4.4 Early Withdrawal of Subjects

4.4.1 When and How to Withdraw Subjects

Participants will be withdrawn from the study at their request (e.g., lack of interest in or perceived need for intervention, lack of time for study assessments).

4.4.2 Data Collection and Follow-up for Withdrawn Subjects

Our team continues to attempt to contact participants for each session and study assessment unless and until they ask us to stop/express the desire to withdraw. Each study contact is an extension of informed consent where participants are told what is occurring, what happens next, and that their participation is voluntary. When we are unable to reach participants by telephone for at least 14 days we send a letter conveying our attempts to reach them and ask them to contact us to continue with study activities or withdraw, as they prefer.

5 Study Intervention

5.1 Description

Theoretical Background of BA/PS

Our approach to improving activity participation reflects self-regulation models that emphasize alignment between goals and circumstances.[55-58] Recognition of a discrepancy between one's goals and circumstances leads to either adaptive or maladaptive coping. Adaptive coping can be viewed as efforts to change the activity, environment, or self. These efforts manifest themselves in active coping (i.e., taking action instead of waiting for problems to disappear), planning (strategically deciding what actions to take), and positive reframing (adjusting expectations and interpretations of events).[29] Goal adjustment is another self-regulation strategy with two components.[59] The first component, goal disengagement, prevents the negative emotional consequences of pursuing a futile goal. The second component, goal reengagement, directs renewed energy towards attainable goals. BA/PS is designed to promote adaptive coping and goal adjustment through a process of strategic goal-setting, problem-solving, and action planning centered on increasing the ease and enjoyment of activity participation and life roles, which leads to lower distress, improved productivity, and higher quality of life.

Figure 3. Behavioral Activation/Problem-solving Framework

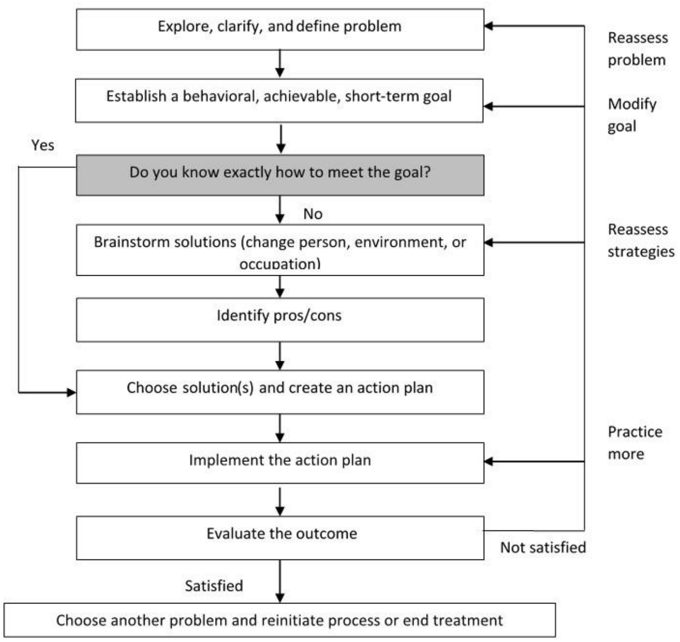

5.2 Treatment Regimen

The BA/PS Intervention (Experimental condition)

Framework (Figure 3). BA/PS teaches survivors to a) systematically examine the reasons an activity is challenging, b) set achievable short-term goals that have the potential to improve participation, c) brainstorm solutions including activity adaptations and

environmental modifications, d) construct and implement a detailed action plan, and e) evaluate the results and level of goal attainment. The structured process gives participants repeated practice in goal reengagement that leads them progressively closer to their long-term functional goals. The BA/PS framework integrates the cognitive-behavioral therapies of Behavioral Activation[60,61] and Problem-solving Treatment[62,63] and incorporates concepts from an occupational therapy theory called the Person-Environment-Occupational Performance Model.[64]

Non-prescriptive. The BA/PS interventionist does not directly give advice or prescriptions. Behavioral Activation and Problem-solving Treatment were developed to treat depression and one active ingredient of those therapies is to teach patients to actively identify their own solutions to problems in living. We have found this technique is also beneficial when addressing participation because the cancer survivor is the expert in his or her lifestyle, routines, and environment. A participant in a pilot study of Behavioral Activation that we are conducting at Johns Hopkins (K23HL138206; PI: Parker) noted this emphasis was refreshing and in stark contrast to his post-hospitalization experiences of rehabilitation (i.e., "they mostly told me what I should do.").

Session 1. The interventionist presents the rationale for BA/PS, promotes a positive problem orientation, and educates about the framework for problem-solving and action planning. The interventionist then administers the Canadian Occupational Performance Measure[65] to elicit participant priorities, motivation, and long-term goals. The interventionist then guides the participant in using the BA/PS framework to set a goal, brainstorm solutions to challenges, and create a detailed action plan for the coming week.

Subsequent sessions (once a week for five more weeks, then once a month for three months). The interventionist begins by reviewing the rationale for BA/PS and the previous action plan and elicits information about goal attainment and satisfaction with effort and outcome. If the goal for that week was met, then the interventionist guides the participant to identify another short-term goal for the coming week that would continue the progress towards the long-term goal. If the goal was not met, then the interventionist troubleshoots with the participant to determine whether the challenge was not fully described, whether the goal was too hard, whether the chosen solution or action plan was not effective, or whether more practice is needed. The framework is then used to create a goal and action plan for the coming week.

Dose. The therapies that BA/PS is built upon typically show that six sessions allow sufficient exposure for participants to meet many of their goals and independently utilize the framework.[36,37] The theoretical rationale for the three monthly sessions comes from the Transtheoretical Model[38] that suggests there is a maintenance stage of intentional behavior change where people need to actively solidify habits and develop confidence in their ability to sustain gains in activity engagement. As such, the three monthly follow-ups are booster sessions to enhance motivation, provide feedback, and keep the focus on continued incremental gains.

Attention Control Condition

Rationale for an attention control. This proposal explores the question, ""Is BA/PS efficacious in enhancing activity participation and quality of life of breast cancer survivors?" Using randomization and a usual care control condition would allow us to eliminate the threats to internal validity that arise with time and history (i.e., participants might improve naturally as time goes by or because of an event occurring at the sites). However, the BA/PS participants would be receiving more attention and support than would the usual care participants. We recognize that the attention experienced within a weekly telephone call from a warm and supportive therapist could lift the spirits of participants and it is theoretically plausible that feeling more hopeful or supported could allow and encourage participants to become more active and engaged in life. We feel it is important to control for this possibility so that we can determine that it is our specific BA/PS intervention and not general support or attention that drives any effects seen in our data.

Rationale for education within the attention control condition. Increased attrition can occur if participants feel that an attention placebo is not a meaningful use of time.[66] As such, we decided to provide education regarding nine cancer survivorship topics (i.e., healthy diets, physical activity, lymphedema management,

smoking cessation, stress management, communication with providers, body image and sexuality, communication with social supports, work accommodations) during the control telephone contacts. The control condition will match the intervention in terms of the number of sessions, the delivery by telephone, use of an occupational therapist, and the use of "homework" between sessions (i.e., reading the education materials for the control condition versus executing the action plan for the BA/PS condition). This will allow us to determine the effect of the specific BA/PS elements (i.e., strategic goal setting, problem-solving, activity adaptation, environmental modification, and action planning) on participation and quality of life.

5.3 Method for Assigning Subjects to Treatment Groups

Scheme. The randomization scheme will be executed via Velos, a clinical trials management software program we have used to facilitate randomization in other studies.[27] Participants will be randomly assigned to group (1:1) using a computer-generated program overseen by Dr Azuero. The randomization scheme will be stratified by site (Dartmouth and Alabama) and time since treatment completion (i.e., <6 months and >6 months) and will be blocked within strata (block lengths of 2 and 4 varied randomly).

Process and blinding. As the Central Coordinating Site, Dartmouth will manage the randomization process. The Dartmouth project manager will be alerted to the assignment by Velos and will trigger the local interventionist to communicate assignment to the participant and initiate treatment activities. All other members of the research team will remain blind to group assignment and participants will be instructed not to discuss their assignment with the Dartmouth research assistant collecting the outcome assessments.

5.4 Preparation and Administration of Study Drug

Not applicable

5.5 Subject Compliance Monitoring

Participation in telephone sessions will be tracked per participant, monitoring the date of the session, number of attempts to reach, and time of day and length of session. For participations in the BA/PS condition, we will track self-reported goal attainment for each 7-day goal that was set.

5.6 Prior and Concomitant Therapy

Not applicable.

5.7 Packaging

Not applicable.

5.8 Blinding of Study Drug

Not applicable.

5.9 Receiving, Storage, Dispensing and Return

5.9.1 Receipt of Drug Supplies

Not applicable.

5.9.2 Storage

Not applicable.

5.9.3 Dispensing of Study Drug

Not applicable.

5.9.4 Return or Destruction of Study Drug

Not applicable.

6 Study Procedures

As the Central Coordinating Site for the study, all data collection will occur from and be managed by Dartmouth staff. A Dartmouth research assistant blind to group assignment will administer standardized telephone interviews, scheduled at the participants' convenience. Data will be directly entered into the Velos software system that our team has used for data collection in many studies. The Velos system is programmed with quality controls that facilitate rigorous data collection, such as not allowing interviewers to skip questions.

Data Collection Schedule

Measures and Data Collection Schedule

Aim	Construct	Instrument	# of Items	T1 Week 0	T2 Week 8	T3 Week 20	T4 Week 44
	Characteristics	Demographics and Clinical Characteristics	9	X			
Aim 1	Participation & Productivity	PROMIS® (Satisfaction and Ability to Participate in Social Roles and Activities)<br>Disability Days and WLQ	16<br><br>25+2	X	X	X	X
Aim 2	Quality of Life	FACT-B	37	X	X	X	X
Exploratory Aim	Adaptive Coping	Brief COPE subscales	6	X	X	X	X
Exploratory Aim	Goal Adjustment	GDGRS	10	X	X	X	X
Exploratory Aim	Distress	PROMIS® Emotional Distress- Depression	8	X	X	X	X

The research assistants will administer the outcome assessment battery by telephone upon enrollment (T1), after completion of the most intensive portion of the intervention (T2), after completion of the full intervention (T3) and six months after completion of the intervention (T4).

Measures (Copies of all measures can be found in the Appendix)

Eligibility Assessment

Participation restrictions: *The Work and Social Adjustment Scale (WSAS)*. The WSAS is a five-item scale of participation restrictions related to work, home management (e.g., cleaning, shopping, childcare), leisure, and relationship activities.[49,67] Items are rated from 0-8 (0 = not at all impaired, 8 = very severely impaired). Test-retest reliability is acceptable at $r = 0.73$. As in the preliminary studies, we will use a WSAS cutoff score of > 10 as a way to identify people with at least a moderate level of participation restrictions.

C.6.3.2 Sociodemographic and Clinical Characteristics to Describe the Sample

Enrolled participants will report their age, race, ethnicity, employment status, education level, marital status, insurance status, number of dependent children living at home, and household income. We will use data from the medical record to describe stage, treatment and co-morbidities.[68]

C.6.3.3 Participation

Participation satisfaction (primary outcome): Participation is a construct with two dimensions: satisfaction and ability. Disability scholars have argued that the subjective assessment of satisfaction with daily routines and activities is a defining feature of participation.[69] As such, we will use the PROMIS *Satisfaction with Social Roles and Activities Short Form 8a*[39,40] as our primary outcome. Initial validation studies used exploratory and confirmatory factor analysis and two-parameter item response theory modeling to explore differential item functioning and to increase the precision of the PROMIS participation items.[39,40] The resulting 8-item subscales (i.e., satisfaction and ability) address routine, work, leisure, family, and social activities. The scales were used in a recent large study of cancer survivors.[47]

Participation ability (secondary outcome): We will use the second 8-item PROMIS scale entitled *Ability to Participate in Social Roles and Activities Short Form 8a*[39,40] to measure the ability aspect of participation. The scale was developed in conjunction with the satisfaction scale, as described above. We added an

item to solicit participant perceptions of the degree to which their challenges are related to cancer or its treatment so that we can determine if the source of the restrictions moderates the effect of the intervention.

Productivity (secondary outcome): We will use relevant questions from the Disability Days section of the *Medical Expenditure Panel Survey (MEPS)* [41,70] to capture days missed from work and lost household productivity. Missed days of work have been defined as half or more of a day missed from work due to a physical illness, or injury, or mental or emotional problem (including those missed because of hospitalizations). MEPS questions can be used to measure missed days of school if survivors are enrolled in school or training. For all participants, including those who are not employed or are on leave, we will assess lost household productivity by measuring days spent in bed, defined in MEPS as half days or more spent in bed because of physical illness or injury, or mental or emotional problems. We will also use the *Work Limitations Questionnaire (WLQ)*[42] to assess productivity. The WLQ has 25 items, and 4 subscales that assess limitations in 4 job dimensions (time, physical, mental/interpersonal/output). The subscale scores represent the percentage of time in the previous 2 weeks that participants may be limited in performing in the specific dimension. An overall WLQ Productivity Loss Score is the weighted sum of the 4 subscale scores and indicates the percentage decrement in productivity. The WLQ demonstrated high validity and reliability[42,71] and has been used in cancer survivor populations.[72-74] While developed to assess productivity at paid work, it has been used with homemakers as well.[75]

C.6.3.4 Quality of Life

Quality of Life: The *Functional Assessment of Cancer Therapy-Breast Cancer (FACT-B)* is a 37-item self-report measure of health-related quality of life specifically designed for breast cancer patients.[43,44] The first 27 items of the tool represent the FACT-G which assesses perceived well-being in physical, social, emotional, and functional domains. The remainder of the tool addresses 10 concerns directly relevant to breast cancer (e.g., self-consciousness about appearance and concerns about weight change and familial patterns of breast cancer). Subscale scores are derived in each domain as well as a total score. The FACT-B has an internal consistency of α=.88.[44] Test-retest reliability demonstrates high correlations at a five-day interval ($r = .97$).

C.6.3.5 Exploratory Outcomes

Coping: *Brief COPE*.[45] Our pilot research has suggested that BA/PS increases participants' use of active coping, planning and positive reframing.[29] These coping styles are measured by three subscales of the Brief COPE. The Brief COPE has been shown to have excellent psychometric properties among cancer patients including evidence of construct, convergent and concurrent criterion validity.[76] To minimize respondent burden, we will not administer the 28-item Brief COPE, but will instead utilize the three subscales of interest.

Goal adjustment: *Goal Disengagement and Goal Reengagement Scale (GDGRS)*.[46] The GDGRS is a 10-item scale that measures two aspects of goal adjustment. Four items measure dispositional goal disengagement (i.e. the general inclination to relinquish untenable goals). Six items measure dispositional goal reengagement (i.e., commit to new goals). The internal consistency of the scales is generally high.[77] We will use the GDGRS to explore the potential efficacy of BA/PS on goal adjustment.

Distress: *PROMIS Emotional Distress- Depression- Short Form 8a.* This PROMIS short form consists of 8 items that measure distressing depressive symptoms. The scale has been used in cancer populations and found to be responsive to patient-perceived worsening or improvement. [47] Because breast cancer survivors can experience significant distress regarding their ability to perform social roles and life activities, we will use this scale to explore the potential efficacy of BA/PS on this outcome.

7 Statistical Plan

7.1 Sample Size Determination

Minimally important differences. The minimally important differences (MID) between the two study arms are taken to be 10% of the practical range of the outcome variable[31] and thus the MIDs are 3.94, 3.95 and 11.2 for satisfaction with participation, ability to participate, and quality of life, respectively. Note that these MIDs are not data dependent, although standard deviations might change for different data sets. We will

assume the standard deviation for the two PROMIS scales are both 10 according to the scoring manual and the standard deviation for FACT-B is 24, as estimated from our third pilot study.[29]

Estimated attrition. To estimate attrition, we examined the attrition seen in our three preliminary studies. One of the preliminary studies was an RCT,[25] two studies used a single arm design,[29] and each of the studies had at least three assessments. On average, 80% of our participants completed all study activities, and thus we expect an attrition of ~20% (if 300 people enroll, at least 240 participants will complete the study).

Power. Using a Time-Averaged Difference approach,[78] at a corrected significance level of .05/3=.017, and with three time-points post baseline, the required sample size to test for the minimally important effects of BA/PS on participation (PROMIS scales) ranges from 63 to 117 in each arm to achieve 80% power for within subject correlations ranging from 0.2-0.8. The required sample size to test for the minimally important effect of BA/PS on quality of life (overall FACT-B score) ranges from 45 to 83 in each arm to achieve 80% power for within subject correlations ranging from 0.2-0.8. An FDR correction (see section C.7.3) will be used at the time of analysis which will provide more power than a Bonferroni correction and adequate control to the number of Type I errors.

7.2 Statistical Methods

Data analysis will begin with descriptive statistics for baseline participant characteristics and outcomes by study group. We will calculate means, standard deviations, and percentiles for continuous variables (e.g., participation, quality of life, coping, goal adjustment, distress, age, etc.) and frequencies and proportions for categorical variables (e.g., clinical characteristics), at each time point as appropriate. We will plot and inspect the distributions of the outcome variables, and examine the validity of any extreme values (data entry errors will be minimized by logic checks in the Velos data collection system, and inspection of scheduled reports during data collection). We will examine balance between study groups with respect to baseline characteristics using effect sizes such as the standardized mean difference for numerical variables and Cramer's V for categorical variables. We will examine patterns of missing data due to dropout, and whether baseline characteristics are associated with dropout. Baseline factors showing non-trivial imbalances between groups or that are predictive of dropout, will be then used as adjusting covariates in the longitudinal group comparisons. We will use the latest versions of standard statistical packages (SAS and R) for all analyses.

Group Comparisons (Aim 1, Aim 2, and Exploratory Aim)

Objective. Our objective is to compare the two groups' functional recovery over time in terms of participation (PROMIS scales; Aim 1), productivity (Disability Days and WLQ; Aim 1), quality of life (FACT-B; Aim 2) and coping style, distress, and goal adjustment (Brief COPE, PROMIS scale and GDGRS; Exploratory Aim). All measures are collected at conceptually relevant time points of enrollment (T1), completion of the most intensive part of the intervention (T2), completion of the full intervention (T3) and six months after treatment completion (T4). The methods described below will be conducted upon each outcome variable.

Modeling. A longitudinal model fitted with linear mixed methods will be used for each outcome. Numerical outcomes with markedly non-normal distributions, if any, will be modeled with more appropriate error distributions than the default normal (e.g., lognormal or generalized beta distributions). The focus of inference will be the between-group difference in outcome trajectories over the study time points, modeled by a time by group interaction. A random effect for subject will be fitted to account for covariance among repeated measures on the same individuals. If necessary, we will conduct covariate adjustment for baseline factors unbalanced between the groups or predictive of dropout. Time will be modeled as a categorical variable, to avoid the strong assumption of linear trajectories, and therefore the single test for the interaction effect will be a multiple-degree of freedom test. Model-predicted outcome means (a.k.a. LS-Means) by group at each time point will be computed to facilitate interpretation. The overall treatment effect will be computed as the between-group difference in change from baseline (change from T1 averaged over T2 to T4) estimated with a linear contrast.

Handling of Missing Data

Mixed modeling techniques and covariate adjustment will reduce the impact of missing data, as the missingness is not assumed completely at random (MCAR) but conditionally (on the covariates) at random (i.e., MAR, a milder assumption).[79] Should dropout exceed the 20% allowed by the sample size (section C.7.5), non-parametric multiple imputation[80] will be employed to determine the robustness of the conclusions for the main analyses under the milder MAR assumption. Because missing data due to non-ignorable or non-random drop out (i.e., MNAR) does not depend on the observed data, it presents the most complex situation to handle. Because of the non-invasive, supportive nature of the BA/PS intervention, as well as the attention control condition, a priori, we do not expect to encounter non-random dropout (and therefore MNAR). However, we will examine the tracking system records and logs with regard to dropout, to determine the main reasons for dropout. If sufficient indication of an MNAR mechanism is found, sensitivity analyses under different assumptions for the missing data mechanism will be conducted, following the methodology described by Molenberghs and Kenward[81] in which the missing data mechanism needs to be modeled explicitly.

Adjustment for Multiple Inference

A False Discovery Rate (FDR) approach[82] will be utilized to adjust inferential results for multiple inferences on the same body of data, separately for the primary analyses and the exploratory analyses. The FDR is the expected proportion of true null differences among those that are declared “significant”. The FDR will be set at 10%. All research products will disclose the number of inferences conducted and whether outcomes were primary or exploratory. As shown in section C.7 5 below, the sample is well powered to detect relevant effect sizes on the primary outcomes, even after multiplicity adjustment.

Heterogeneity of Intervention Effects

Rationale. We have developed the intervention to be flexible and responsive to the needs of a diverse group of breast cancer survivors. However, our diverse sample will give us the opportunity to identify any unanticipated moderator effects. Therefore, we will explore whether there are subgroups of participants who benefited most and least during the study, as per the primary outcomes.

Recursive partitioning. The classical approach to moderator or subgroup analysis consists of 3 steps: 1) pre-specifying some population characteristics of interest (e.g., site: UAB vs. Dartmouth; race: minority vs. white; age: <65 vs. ≥65; source of participation restriction: primarily related to or unrelated to cancer; relapse vs. not, etc.); 2) analyzing one characteristic at a time, conducting inferences on differential intervention effects based on the characteristic's subgroups, using interaction tests adjusted for subgroup imbalances in other characteristics; and 3) applying an adjustment for multiple inference to account for the multiple analyses on the same body of data. Instead of implementing the classical approach, we propose using recursive partitioning, a.k.a. CART[83] (Classification and Regression Trees), a non-parametric modeling approach that allows extracting multivariate profiles from a sufficiently large dataset under minimal modeling assumptions, based on values of an outcome and participant characteristics. We propose this approach instead of the classical approach because multiple patient characteristics might be simultaneously associated with benefitting from the BA/PS intervention, therefore the multivariate approach can potentially provide more information and be more useful than the classical approach. Because CART is data-driven, these analyses will be considered exploratory. To avoid assuming that missing outcome data due to dropout is missing completely at random (i.e., MCAR), a Random Forest-based algorithm[80,83] will be used to generate 3 imputed datasets comprising baseline characteristics, group assignment, and longitudinal outcome variables. CART modeling will be implemented on each imputed dataset and results will be compared as a form of sensitivity analysis. The target variables for the CART modeling will be the average change from baseline in each primary outcome. Regression tree models for these target variables will be fitted using as predictors the group assignment and selected baseline characteristics. These baseline characteristics will include indicators of pertinent subpopulations of interest: site, race, income, age, etc. We will implement the conditional inference approach[84] to fit the tree models and use repeated 10-fold cross-validation to determine the final tree size. The tree model is a

decision-tree-like structure that is interpreted based on the characteristics of the resulting groups of participants.

7.3 Subject Population(s) for Analysis

Intent to treat. Our main analyses will utilize an intention-to-treat approach. As the BA/PS intervention is not part of standard practice, cross-over events are not expected to occur. All available data from all participants who undergo randomization will be included in the analyses according to the group assigned, regardless of any post-randomization protocol deviations.

8 Safety and Adverse Events

8.1 Definitions

Unanticipated Problems Involving Risk to Subjects or Others
Any incident, experience, or outcome that meets all of the following criteria:

- Unexpected in nature, severity, or frequency (i.e. not described in study-related documents such as the IRB-approved protocol or consent form, the investigators brochure, etc)
- Related or possibly related to participation in the research (i.e. possibly related means there is a reasonable possibility that the incident experience, or outcome may have been caused by the procedures involved in the research)
- Suggests that the research places subjects or others at greater risk of harm (including physical, psychological, economic, or social harm).

Adverse Event
An ***adverse event*** (AE) is any symptom, sign, illness or experience that develops or worsens in severity during the course of the study. Intercurrent illnesses or injuries should be regarded as adverse events. Abnormal results of diagnostic procedures are considered to be adverse events if the abnormality:

- results in study withdrawal
- is associated with a serious adverse event
- is associated with clinical signs or symptoms
- leads to additional treatment or to further diagnostic tests
- is considered by the investigator to be of clinical significance

Serious Adverse Event
Adverse events are classified as serious or non-serious. A ***serious adverse event*** is any AE that is:

- fatal
- life-threatening
- requires or prolongs hospital stay
- results in persistent or significant disability or incapacity
- a congenital anomaly or birth defect
- an important medical event

Important medical events are those that may not be immediately life threatening, but are clearly of major clinical significance. They may jeopardize the subject, and may require intervention to prevent one of the other serious outcomes noted above. For example, drug overdose or abuse, a seizure that did not result in in-patient hospitalization, or intensive treatment of bronchospasm in an emergency department would typically be considered serious.

All adverse events that do not meet any of the criteria for serious should be regarded as ***non-serious adverse events***.

Adverse Event Reporting Period
The study period during which adverse events must be reported is normally defined as the period from the initiation of any study procedures to the end of the study treatment follow-up.

Preexisting Condition
A preexisting condition is one that is present at the start of the study. A preexisting condition should be recorded as an adverse event if the frequency, intensity, or the character of the condition worsens during the study period.

General Physical Examination Findings
At screening, any clinically significant abnormality should be recorded as a preexisting condition. At the end of the study, any new clinically significant findings/abnormalities that meet the definition of an adverse event must also be recorded and documented as an adverse event.

Post-study Adverse Event
All unresolved adverse events should be followed by the investigator until the events are resolved, the subject is lost to follow-up, or the adverse event is otherwise explained. At the last scheduled visit, the investigator should instruct each subject to report any subsequent event(s) that the subject, or the subject's personal physician, believes might reasonably be related to participation in this study. The investigator should notify the study sponsor of any death or adverse event occurring at any time after a subject has discontinued or terminated study participation that may reasonably be related to this study.

Abnormal Laboratory Values
A clinical laboratory abnormality should be documented as an adverse event if any one of the following conditions is met:

- The laboratory abnormality is not otherwise refuted by a repeat test to confirm the abnormality
- The abnormality suggests a disease and/or organ toxicity
- The abnormality is of a degree that requires active management; e.g. change of dose, discontinuation of the drug, more frequent follow-up assessments, further diagnostic investigation, etc.

Hospitalization, Prolonged Hospitalization or Surgery
Any adverse event that results in hospitalization or prolonged hospitalization should be documented and reported as a serious adverse event unless specifically instructed otherwise in this protocol. Any condition responsible for surgery should be documented as an adverse event if the condition meets the criteria for and adverse event.

Neither the condition, hospitalization, prolonged hospitalization, nor surgery are reported as an adverse event in the following circumstances:

- Hospitalization or prolonged hospitalization for diagnostic or elective surgical procedures for a preexisting condition. Surgery should ***not*** be reported as an outcome of an adverse event if the purpose of the surgery was elective or diagnostic and the outcome was uneventful.
- Hospitalization or prolonged hospitalization required to allow efficacy measurement for the study.
- Hospitalization or prolonged hospitalization for therapy of the target disease of the study, unless it is a worsening or increase in frequency of hospital admissions as judged by the clinical investigator.

8.2 Recording of Adverse Events

At each contact with the subject, the investigator must seek information on adverse events by specific questioning and, as appropriate, by examination. Information on all adverse events should be recorded immediately in the source document, and also in the appropriate adverse event module of the case report form (CRF). All clearly related signs, symptoms, and abnormal diagnostic procedures results should recorded in the source document, though should be grouped under one diagnosis.

All adverse events occurring during the study period must be recorded. The clinical course of each event should be followed until resolution, stabilization, or until it has been determined that the study treatment or participation is not the cause. Serious adverse events that are still ongoing at the end of the study period must be followed up to determine the final outcome. Any serious adverse event that occurs after the

study period and is considered to be possibly related to the study treatment or study participation should be recorded and reported immediately.

8.3 Reporting of Serious Adverse Events and Unanticipated Problems

Investigators must conform to the adverse event reporting timelines, formats and requirements of the various entities to which they are responsible, but at a minimum those events that must be reported are those that are:

- related to study participation,
- unexpected, and
- serious or involve risks to subjects or others
  (see definitions, section 8.1).

If the report is supplied as a narrative, the minimum necessary information to be provided at the time of the initial report includes:

- Study identifier
- Study Center
- Subject number
- A description of the event
- Date of onset
- Current status
- Whether study treatment was discontinued
- The reason why the event is classified as serious
- Investigator assessment of the association between the event and study treatment

8.3.1 Investigator reporting: notifying the study sponsor

Not applicable.

8.3.2 Investigator reporting: notifying the Dartmouth IRB

This section describes the requirements for safety reporting by investigators who are Dartmouth faculty, affiliated with a Dartmouth research site, or otherwise responsible for safety reporting to the Dartmouth IRB. The Dartmouth IRB requires reporting of those events related to study participation that are unforeseen and indicate that participants or others are at increased risk of harm. The Dartmouth IRB requires researchers to submit reports of *any incident, experience, or outcome that meets each of the following criteria:*

- ***Unanticipated*** *in terms of nature, severity, or frequency given: (a) the research procedures that are described in the protocol-related documents, such as the IRB-approved research protocol and consent document; and (b) the characteristics of the subject population being studied; and*
- ***Possibly related*** *to participation in the research means there is a reasonable possibility that the incident, experience, or outcome may have been associated with research participation; and*
- *The problem suggests that the research places subjects or others at a* ***greater risk of harm*** *(including physical, psychological, emotional, economic, legal, or social harms) than was previously known or recognized.*

Reporting Process

Unanticipated problems posing risks to subjects or others as noted above will be reported to the Dartmouth IRB using the form: “Unanticipated Problem Involving Risks to Subjects or Others (UPR).”

Copies of each report and documentation of IRB notification and receipt will be kept in the Clinical Investigator’s study file.

Other Reportable events:

For clinical trials, the following events are also reportable to the Dartmouth IRB:

- Any adverse experience, defined as an untoward or unfavorable medical occurrence in a human subject, including any abnormal sign (for example, abnormal physical exam or laboratory finding), symptom, or disease, temporally associated with the subject’s participation in research, whether or not considered related to the subject’s participation in the research), that is considered:

  - Serious: Death; a life-threatening adverse drug experience; inpatient hospitalization or prolongation of existing hospitalization; a persistent or significant disability or incapacity; or a congenital anomaly or birth defect; and
  - Unexpected: Any adverse experience, the specificity or severity of which is not consistent with the current investigator brochure or consent form; and
  - Possibly related: There is a reasonable possibility that the incident, experience, or outcome may have been associated with the procedures involved in the research; and
  - Is experienced by a participant in a trial open at a site subject to Dartmouth IRB review
- Information that indicates a change to the risks or potential benefits of the research, in terms of severity or frequency. For example:
  - An interim analysis indicates that participants have a lower rate of response to treatment than initially expected.
  - Safety monitoring indicates that a particular side effect is more severe, or more frequent than initially expected.
  - A paper is published from another study that shows that an arm of your research study is of no therapeutic value.
- Change in FDA safety labeling or withdrawal from marketing of a drug, device, or biologic used in a research protocol.
- Breach of confidentiality
- Change to the protocol taken without prior IRB review to eliminate apparent immediate hazard to a research participant.
- Complaint of a participant when the complaint indicates unexpected risks or the complaint cannot be resolved by the research team.
- Protocol deviation (meaning an accidental or unintentional deviation from the IRB approved protocol) that in the opinion of the investigator placed one or more participants at increased risk, or affects the rights or welfare of subjects.

8.3.3 Investigator reporting: Notifying a non-Dartmouth IRB

Not applicable (Dartmouth will serve as the IRB of record for this mulit-site study).

8.3.4 Sponsor reporting: Notifying the FDA

Not applicable

8.3.5 Sponsor reporting: Notifying participating investigators

It is the responsibility of the study PI to notify all participating investigators of any adverse event associated with the study.

8.4 Unblinding Procedures

Not applicable.

8.5 Stopping Rules

Significant risk is not anticipated in this study due to the supportive and non-invasive nature of the intervention. However, we will summarize adverse event data annually to the DSMB for consideration of study continuation. The study design does not include planned interim analyses to identify the need to stop early due to significant benefit, i.e., inferential analyses will be conducted when data collection has been completed.

8.6 Medical Monitoring

Not applicable.

8.6.1 Internal Data and Safety Monitoring Board

This project will involve enrolling 300 breast cancer survivors into a randomized controlled trial within the year after they complete their treatment. After enrollment, participants will complete a baseline assessment. Participants randomized to the BA/PS intervention arm will engage in 9 individual sessions delivered via telephone by an occupational therapist. Participants randomized to the attention control arm will engage in 9 individual education sessions delivered via telephone by an occupational therapist. The intervention and assessments are not invasive and do not involve pharmacological agents. The informed consent process, the recruitment process, and the timeliness and quality of the data will be monitored by the principal investigator, the Institutional Review Board, and the Data Safety Monitoring and Accrual Committee (DSMAC) of the Norris Cotton Cancer Center. The DSMAC meets quarterly to review accrual rates and information for studies that have accrued participants. The Clinical Cancer Review Committee (CCRC) determines the frequency of DSMAC review. The DSMAC has the authority to suspend or to recommend termination to the CCRC of all research activities that fall within its jurisdiction. In the event that a study is suspended or terminated, that information will be forwarded to the CPHS (Dartmouth IRB) office.

.

The DSMC has established procedures of operation and guidelines for determining the methods and frequency for review of departmental studies. Based on the DSMC's established guidelines, the proposed study's score of 5 on the 10-point scoring system indicates that the principal monitor for the study will be the PI (Dr. Lyons) and she will present the DSMC with a summary of the accumulated data and safety information during an annual review for each year of the study.

8.6.2 Independent Data and Safety Monitoring Board

Not applicable.

9 Data Handling and Record Keeping

9.1 Confidentiality

Information about study subjects will be kept confidential and managed according to the requirements of the Health Insurance Portability and Accountability Act of 1996 (HIPAA). Those regulations require a signed subject authorization informing the subject of the following:

- What protected health information (PHI) will be collected from subjects in this study
- Who will have access to that information and why
- Who will use or disclose that information
- The rights of a research subject to revoke their authorization for use of their PHI.

In the event that a subject revokes authorization to collect or use PHI, the investigator, by regulation, retains the ability to use all information collected prior to the revocation of subject authorization. For subjects that have revoked authorization to collect or use PHI, attempts should be made to obtain permission to collect at least vital status (i.e. that the subject is alive) at the end of their scheduled study period.

9.2 Source Documents

Source data is all information, original records of clinical findings, observations, or other activities in a clinical trial necessary for the reconstruction and evaluation of the trial. Source data are contained in source documents. Examples of these original documents, and data records include: hospital records, clinical and office charts, laboratory notes, memoranda, subjects' diaries or evaluation checklists,

pharmacy dispensing records, recorded data from automated instruments, copies or transcriptions certified after verification as being accurate and complete, microfiches, photographic negatives, microfilm or magnetic media, x-rays, subject files, and records kept at the pharmacy, at the laboratories, and at medico-technical departments involved in the clinical trial.

9.3 Case Report Forms

The study case report form (CRF) is the primary data collection instrument for the study. All data requested on the CRF must be recorded. All missing data must be explained. If a space on the CRF is left blank because the procedure was not done or the question was not asked, write “N/D”. If the item is not applicable to the individual case, write “N/A”. All entries should be printed legibly in black ink. If any entry error has been made, to correct such an error, draw a single straight line through the incorrect entry and enter the correct data above it. All such changes must be initialed and dated. DO NOT ERASE OR WHITE OUT ERRORS. For clarification of illegible or uncertain entries, print the clarification above the item, then initial and date it.

9.4 Records Retention

We will store records for at least 10 years after the completion of the study.

10 Study Monitoring, Auditing, and Inspecting

10.1 Study Monitoring Plan

Drs. Lyons and Bakitas have developed and utilized structured processes for ensuring fidelity to study protocols including a) a core training and study-specific refresher course in standardized interviewing that includes role playing and supervision, b) frequent (at least every other week) team meetings to troubleshoot recruitment and data collection issues, and c) use of data entry software that provides entry validation range tests and is designed to minimize missing data by flagging missed items before surveys are submitted. Research assistants will manually extract data from the medical record regarding the participants’ clinical characteristics of stage, treatment, and co-morbidities. To ensure accuracy, the assistants will have site-specific training to identify the location of the information in the medical record and we will audit a random 10% of the medical records to verify the accuracy of the abstracted information. The project manager at Dartmouth will maintain and monitor the database regularly throughout the study. The database is password-protected and sits behind the firewall of the Norris Cotton Cancer Center.

10.2 Auditing and Inspecting

Not applicable.

11 Ethical Considerations

This study is to be conducted according to US and international standards of Good Clinical Practice (FDA Title 21 part 312 and International Conference on Harmonization guidelines), applicable government regulations and Institutional research policies and procedures.

This protocol and any amendments will be submitted to a properly constituted independent Ethics Committee (EC) or Institutional Review Board (IRB), in agreement with local legal prescriptions, for formal approval of the study conduct. The decision of the EC/IRB concerning the conduct of the study will be made in writing to the investigator and a copy of this decision will be provided to the sponsor before commencement of this study. The investigator should provide a list of EC/IRB members and their affiliate to the sponsor.

All subjects for this study will be provided a consent form describing this study and providing sufficient information for subjects to make an informed decision about their participation in this study. See

Attachment for a copy of the Subject Informed Consent Form. This consent form will be submitted with the protocol for review and approval by the EC/IRB for the study. The formal consent of a subject, using the EC/IRB-approved consent form, must be obtained before that subject undergoes any study procedure. The consent form must be signed by the subject or legally acceptable surrogate, and the investigator-designated research professional obtaining the consent.

12 Study Finances

12.1 Funding Source

The study will not be conducted unless funding is awarded by the National Cancer Institute. Council review is pending in October of 2018.

12.2 Conflict of Interest

Any investigator who has a conflict of interest with this study (patent ownership, royalties, or financial gain greater than the minimum allowable by their institution, etc.) must have the conflict reviewed by a properly constituted Conflict of Interest Committee with a Committee-sanctioned conflict management plan that has been reviewed and approved by the study sponsor prior to participation in this study. All Dartmouth-Hitchcock investigators will follow the Dartmouth-Hitchcock conflict of interest policy.

12.3 Subject Stipends or Payments

Participants will be paid $20 for completion of each of the Time 1, Time 2, and Time 3 assessments and will be paid $30 upon completion of the Time 4 assessment (total = $90 per participant).

13 Publication Plan

The PI and co-investigators will prospectively register the trial in clinicaltrials.gov and will be responsible for co-authoring publishing the results of study.

14 References

15 Attachments

Appendix A: Treatment Manual for BA/PS
Appendix B: Measures

1. World Health Organization. International Classification of Functioning, Disability, and Health (ICF). In. Geneva, Switzerland: World Health Organization; 2002.
2. Chang F-H, Coster WJ. Conceptualizing the Construct of Participation in Adults With Disabilities. *Arch Phys Med Rehabil.* 2014;95(9):1791-1798.
3. Chang F-H, Chang K-H, Liou T-H, Whiteneck GG. Validation of the Participation Measure–3 Domains, 4 Dimensions (PM-3D4D). *Arch Phys Med Rehabil.* 2017;98(12):2498-2506.
4. Ness KK, Wall MM, Oakes JM, Robison LL, Gurney JG. Physical performance limitations and participation restrictions among cancer survivors: a population-based study. *Ann Epidemiol.* 2006;16(3):197-205.
5. Letellier M-E, Mayo N. Assessment of breast cancer disability: agreement between expert assessment and patient reports. *Disabil Rehabil.* 2017;39(8):798-808.
6. Yang EJ, Kang E, Kim S-W, Lim J-Y. Discrepant trajectories of impairment, activity, and participation related to upper-limb function in patients with breast cancer. *Arch Phys Med Rehabil.* 2015;96(12):2161-2168.
7. Jones JM, Olson K, Catton P, et al. Cancer-related fatigue and associated disability in post-treatment cancer survivors. *J Cancer Surviv.* 2016;10(1):51-61.

8. Deimling GT, Pappada H, Ye M, et al. Factors Affecting Perceptions of Disability and Self-Rated Health Among Older Adult, Long-Term Cancer Survivors. *J Aging Health.* 2017;0(0):0898264317745745.
9. Kenzik KM, Kent EE, Martin MY, Bhatia S, Pisu M. Chronic condition clusters and functional impairment in older cancer survivors: a population-based study. *J Cancer Surviv.* 2016;10(6):1096-1103.
10. Braithwaite D, Satariano WA, Sternfeld B, et al. Long-term prognostic role of functional limitations among women with breast cancer. *J Natl Cancer Inst.* 2010;102(19):1468-1477.
11. DiSipio T, Hayes S, Battistutta D, Newman B, Janda M. Patterns, correlates, and prognostic significance of quality of life following breast cancer. *Psychooncology.* 2011;20(10):1084-1091.
12. Sehl M, Lu X, Silliman R, Ganz P. Decline in physical functioning in first 2 years after breast cancer diagnosis predicts 10-year survival in older women. *J Cancer Surviv.* 2013;7(1):20-31.
13. Williamson GM. Extending the activity restriction model of depressed affect: Evidence from a sample of breast cancer patients. *Heath Psychology.* 2000;19(4):339-347.
14. Low CA, Stanton AL. Activity disruption and depressive symptoms in women living with metastatic breast cancer. *Health Psychol.* 2015;34(1):89-92.
15. Feuerstein M. Cancer survivors need evidence on how to optimize physical function. *J Cancer Surviv.* 2009;3(2):73-74.
16. Nekhlyudov L, Ganz PA, Arora NK, Rowland JH. Going Beyond Being Lost in Transition: A Decade of Progress in Cancer Survivorship. *J Clin Oncol.* 2017;38(18):1978-1981.
17. Egan MY, McEwen S, Sikora L, Chasen M, Fitch M, Eldred S. Rehabilitation following cancer treatment. *Disabil Rehabil.* 2013;35(26):2245-2258.
18. Howell D, Harth T, Brown J, Bennett C, Boyko S. Self-management education interventions for patients with cancer: a systematic review. *Support Care Cancer.* 2017;25(4):1323-1355.
19. Cheng KKF, Lim YTE, Koh ZM, Tam WWS. Home-based multidimensional survivorship programmes for breast cancer survivors. *Cochrane Database Syst Rev.* 2017(8).
20. Zhu G, Zhang X, Wang Y, Xiong H, Zhao Y, Sun F. Effects of exercise intervention in breast cancer survivors: a meta-analysis of 33 randomized controlled trials. *Onco Targets Ther.* 2016;9:2153-2168.
21. Cella DF, Tulsky DS, Gray G, et al. The Functional Assessment of Cancer Therapy scale: development and validation of the general measure. *J Clin Oncol.* 1993;11(3):570-579.
22. Cheville AL, Kornblith AB, Basford JR. An examination of the causes for the underutilization of rehabilitation services among people with advanced cancer. *Am J Phys Med Rehabil.* 2011;90(5 Suppl 1):S27-37.
23. Campbell KL, Pusic AL, Zucker DS, et al. A prospective model of care for breast cancer rehabilitation: Function. *Cancer.* 2012;118(S8):2300-2311.
24. American Occupational Therapy Association. Occupational Therapy Practice Framework: Domain and Process (3rd Edition). *Am J Occup Ther.* 2014;68(Supplement_1):S1-S48.
25. Hegel MT, Lyons KD, Hull JG, et al. Feasibility study of a randomized controlled trial of a telephone-delivered problem-solving-occupational therapy intervention to reduce participation restrictions in rural breast cancer survivors undergoing chemotherapy. *Psychooncology.* 2011;20(10):1092-1101.
26. Bakitas MA, Lyons KD, Hegel MT, et al. The Project ENABLE II randomized controlled trial to improve palliative care for rural patients with advanced cancer: Baseline findings, methodological challenges, and solutions. *Palliative and Supportive Care.* 2009;7:75-86.
27. Bakitas MA, Tosteson TD, Li Z, et al. Early versus delayed initiation of concurrent palliative oncology care: Patient outcomes in the ENABLE III randomized controlled trial. *J Clin Oncol.* 2015;33(13):1438-1445.
28. Lyons KD, Newman R, Adachi-Mejia AM, Whipple J, Hegel MT. Content Analysis of a Participant-Directed Intervention to Optimize Activity Engagement of Older Adult Cancer Survivors. *OTJR: Occupation, Participation and Health.* 2018;38(1):38-45.
29. Lyons KD, Hull JG, Kaufman PA, et al. Development and initial evaluation of a telephone-delivered, Behavioral Activation and Problem-solving Treatment Program to address functional goals of breast cancer survivors *J Psychosoc Oncol.* 2015;33(2):199-218.
30. Cella D, Hahn EA, Dineen K. Meaningful change in cancer-specific quality of life scores: Differences between improvement and worsening. *Qual Life Res.* 2002;11:207-221.

31. Ringash J, O'Sullivan B, Bezjak A, Redelmeier DA. Interpreting clinically significant changes in patient-reported outcomes. *Cancer.* 2007;110(1):196-202.
32. Lyons KD, Erickson KS, Hegel MT. Problem-solving strategies of women undergoing chemotherapy for breast cancer. *Canadian Journal of Occupational Therapy.* 2012;79(1):33-40.
33. Lyons KD, Svensborn IA, Kornblith AB, Hegel MT. A content analysis of recovery strategies of breast cancer survivors enrolled in a goal-setting intervention. *OTJR: Occupation, Participation and Health.* 2015;35(2):73-80.
34. Bakitas MA, Lyons KD, Hegel MT, et al. Effects of a palliative care intervention on clinical outcomes in patients with advanced cancer: The Project ENABLE II randomized controlled trial. *JAMA.* 2009;302(7):741-749.
35. Bakitas MA, Stevens M, Ahles T, et al. Project ENABLE: A palliative care demonstration project for advanced cancer patients in three settings. *J Palliat Med.* 2004;7(2):363-372.
36. Rovner BW, Casten RJ, Hegel MT, et al. Low vision depression prevention trial in age-related macular degeneration: A randomized clinical trial. *Ophthalmology.* 2014;121(11):2204-2211.
37. Arean P, Hegel M, Vannoy S, Fan M-Y, Unuzter J. Effectiveness of Problem-Solving Therapy for older, primary care patients with depression: Results from the IMPACT Project. *The Gerontologist.* 2008;48(3):311-323.
38. Prochaska JO, Redding CA, Evers KE. The Transtheoretical Model and Stages of Change. In: Glanz K, Rimer BK, Viswanath K, eds. *Health Behavior: Theory, Research and Practice.* 5th ed. San Francisco: Jossey-Bass; 2015:125-148.
39. Castel LD, Williams KA, Bosworth HB, et al. Content validity in the PROMIS social-health domain: a qualitative analysis of focus-group data. *Qual Life Res.* 2008;17(5):737-749.
40. Hahn EA, Devellis RF, Bode RK, et al. Measuring social health in the patient-reported outcomes measurement information system (PROMIS): item bank development and testing. *Qual Life Res.* 2010;19(7):1035-1044.
41. Zheng Z, Yabroff KR, Guy GP, Jr., et al. Annual medical expenditure and productivity loss among colorectal, female breast, and prostate cancer survivors in the United States. *J Natl Cancer Inst.* 2016;108(5).
42. Lerner D, Reed JI, Massarotti E, Wester LM, Burke TA. The Work Limitations Questionnaire's validity and reliability among patients with osteoarthritis. *J Clin Epidemiol.* 2002;55(2):197-208.
43. Brady MJ, Cella DF, Mo F, et al. Reliability and validity of the functional assessment of cancer therapy - Breast Quality-of-Life instrument. *J Clin Oncol.* 1997;15(3):974-986.
44. Coster S, Poole K, Fallowfield LJ. The validation of a quality of life scale to assess the impact of arm morbidity in breast cancer patients post-operatively. *Breast Cancer Res Treat.* 2001;68(3):273-282.
45. Carver CS. You want to measure coping but your protocol's too long: consider the brief COPE. *Int J Behav Med.* 1997;4(1):92-100.
46. Wrosch C, Scheier MF, Miller GE, Schulz R, Carver CS. Adaptive self-regulation of unattainable goals: goal disengagement, goal reengagement, and subjective well-being. *Personality and Social Psychology Bulletin.* 2003;29(12):1494-1508.
47. Jensen RE, Moinpour CM, Potosky AL, et al. Responsiveness of 8 Patient-Reported Outcomes Measurement Information System (PROMIS) measures in a large, community-based cancer study cohort. *Cancer.* 2017;123(2):327-335.
48. Hammel J. A systematic literature review of participation-focused interventions for people with physical disabilities: American Occupational Therapy Foundation (AOTF) Research Colloquium. American Occupational Therapy Association Annual Conference and Expo; April 8, 2016, 2016; Chicago, IL.
49. Mundt JC, Marks IM, Shear MK, Greist JH. The Work and Social Adjustment Scale: a simple measure of impairment in functioning. *Br J Psychiatry.* 2002;180:461-464.
50. Leach CR, Weaver KE, Aziz NM, et al. The complex health profile of long-term cancer survivors: prevalence and predictors of comorbid conditions. *J Cancer Surviv.* 2015;9(2):239-251.
51. Alfano CM, Ganz PA, Rowland JH, Hahn EE. Cancer survivorship and cancer rehabilitation: revitalizing the link. *J Clin Oncol.* 2012;30(9):904-906.
52. American Cancer Society. *Cancer Treatment and Survivorship Facts and Figures 2014-2015.* Atlanta: American Cancer Society; 2015.

53. Callahan CM, Unverzagt FW, Hui SL, Perkins AJ, Hendrie HC. Six-item screener to identify cognitive impairment among potential subjects for clinical research. *Med Care.* 2002;40(9):771-781.
54. Ahles TA, Root JC, Ryan EL. Cancer- and cancer treatment–associated cognitive change: An update on the state of the science. *J Clin Oncol.* 2012;30(30):3675-3686.
55. Carver CS, Scheier MF. *On the self-regulation of behavior.* New York, NY, US: Cambridge University Press; 1998.
56. Carver CS, Smith RG, Petronis VM, Antoni MH. Quality of life among long-term survivors of breast cancer: different types of antecedents predict different classes of outcomes. *Psychooncology.* 2006;15(9):749-758.
57. Carver CS, Pozo C, Harris SD, et al. How coping mediates the effect of optimism on distress: A study of women with early stage breast cancer. *J Pers Soc Psychol.* 1993;65(2):375-390.
58. Hull J. Modeling the Structure of Self-Knowledge and the Dynamics of Self-Regulation. In: Tesser A, Stapel DA, Wood JV, eds. *Self and Motivation: Emerging Psychological Perspectives.* Washington, DC: American Psychological Association; 2002:173-206.
59. Wrosch C, Sabiston CM. Goal adjustment, physical and sedentary activity, and well-being and health among breast cancer survivors. *Psychooncology.* 2013;22(3):581-589.
60. Hopko DR, Lejuez CW, Ruggiero KJ, Eifert GH. Contemporary behavioral activation treatments for depression: Procedures, principles, and progress. *Clin Psychol Rev.* 2003;23(5):699-717.
61. Cuijpers P, van Straten A, Warmerdam L. Behavioral activation treatments of depression: A meta-analysis. *Clin Psychol Rev.* 2007;27(3):318-326.
62. Cuijpers P, van Straten A, Warmerdam L. Problem solving therapies for depression: A meta-analysis. *Eur Psychiatry.* 2007;22(1):9-15.
63. Hegel MT, Arean PA. *Problem-solving treatment for primary care: A treatment manual for depression, Project IMPACT.* Hanover, NH: Dartmouth College; 2003.
64. Baum CM, Christiansen CH. Person-environment-occupation-performance: An occupation-based framework for practice. In: Christiansen CH, Baum CM, Bass-Haugen J, eds. *Occupational therapy: Performance, participation, and well-being.* Thorofare, NJ: SLACK, Inc; 2005:243-266.
65. Law M, Baptiste S, Carswell A, McColl MA, Polatajko H, Pollock N. *Canadian Occupational Performance Measure.* 5th ed. Toronto: Canadian Association of Occupational Therapists; 2014.
66. Popp L, Schneider S. Attention placebo control in randomized controlled trials of psychosocial interventions: theory and practice. *Trials.* 2015;16:150.
67. Marks IM. *Behavioural Psychotherapy; Maudsley Pocketbook of Clinical Management.* Bristol1986.
68. Charlson M, Pompei P, Ales K, MacKenzie C. A new method of classifying prognostic comorbidity in longitudinal studies: Development and validation. *J Chronic Dis.* 1987;40:373-383.
69. Martin Ginis KA, Evans MB, Mortenson WB, Noreau L. Broadening the conceptualization of participation of persons with physical disabilities: A configurative review and recommendations. *Arch Phys Med Rehabil.* 2017;98(2):395-402.
70. Ekwueme DU, Yabroff KR, Guy GP, Jr., et al. Medical costs and productivity losses of cancer survivors--United States, 2008-2011. *MMWR Morb Mortal Wkly Rep.* 2014;63(23):505-510.
71. Lerner D, Amick BC, 3rd, Lee JC, et al. Relationship of employee-reported work limitations to work productivity. *Med Care.* 2003;41(5):649-659.
72. Tamminga SJ, Verbeek JH, Frings-Dresen MH, De Boer AG. Measurement properties of the Work Limitations Questionnaire were sufficient among cancer survivors. *Qual Life Res.* 2014;23(2):515-525.
73. Leensen MCJ, Groeneveld IF, Heide IV, et al. Return to work of cancer patients after a multidisciplinary intervention including occupational counselling and physical exercise in cancer patients: a prospective study in the Netherlands. *BMJ Open.* 2017;7(6):e014746.
74. Feuerstein M, Hansen JA, Calvio LC, Johnson L, Ronquillo JG. Work productivity in brain tumor survivors. *J Occup Environ Med.* 2007;49(7):803-811.
75. Kennedy M, Papneja A, Thavaneswaran A, Chandran V, Gladman DD. Prevalence and predictors of reduced work productivity in patients with psoriatic arthritis. *Clin Exp Rheumatol.* 2014;32(3):342-348.
76. Fillion L, Kovacs A, Gagnon P, Endler N. Validation of the Shortened COPE for use with Breast Cancer Patients Undergoing Radiation Therapy. *Current Psychology.* 2002;21(1):17-34.

77. Thompson EH, Stanton AL, Bower JE. Situational and dispositional goal adjustment in the context of metastatic cancer. *J Pers.* 2013;81(5):441-451.
78. Brown H, Prescott R. *Applied Mixed Models in Medicine, 3rd Ed.* Chichester, England: Wiley; 2015.
79. van Buuren S. *Flexible imputation of missing data.* Boca Raton, FL: CRC Press; 2012.
80. van Buuren S, Groothuis-Oudshoorn K. mice: Multivariate Imputation by Chained Equations in R. *Journal of Statistical Software; Vol 1, Issue 3 (2011).* 2011.
81. Molenberghs G, Kenward M. *Missing Data in Clinical Studies.* Hoboken: Wiley; 2007.
82. Benjamini Y, Hochberg Y. Controlling the false discovery rate: A practical and powerful approach to multiple testing. *Journal of the Royal Statistical Society Series B (Methodological).* 1995;57(1):289-300.
83. Strobl C, Malley J, Tutz G. An introduction to recursive partitioning: rationale, application, and characteristics of classification and regression trees, bagging, and random forests. *Psychol Methods.* 2009;14(4):323-348.
84. Hothorn T, Hornik K, Zeileis A. Unbiased recursive partitioning: A conditional inference framework. *Journal of Computational and Graphical Statistics.* 2006;15(3):651-674.
85. Jennison C, Turnbull B. *Group Sequential Methods with Applications to Clinical Trials.* Boca Raton, FL: Chapman & Hall/CRC Press; 2000.

Protocol Changes for the Optimizing Functional Recovery Randomized Clinical Trial
Note. Grant funding began in February 2019, trial began enrolling in August 2019, and the protocol paper was published in February 2020)

Protocol Version	Protocol Date	Summary of Changes
1	7/18/18	Not applicable- original protocol
2	2/8/19	Changes to measures:<br>• Use Work Limitations Questionnaire-short form instead of Work Limitations Questionnaire to reduce burden<br>• Use FACT-General instead of FACT-Breast to reduce burden<br>• Use Hospital Anxiety and Depression scale instead of PROMIS-Emotional Distress-Depression to capture anxiety in addition to depression<br>• Added five questions about perceived benefits as a manipulation check<br>• Added individual activity targets to Aim 1 measurement to use an individualized outcome measure of participation restrictions<br>Change to staff: Hiring coordinators instead of Recruitment and Retention Core of University of Alabama at Birmingham<br>Added another strata to randomization scheme: received chemotherapy versus did not receive chemotherapy<br>Funding source updated and participant reimbursement for survey completion increased by $5 at three time points (from $90 to $105 for total data completion).
3	10/4/19	Updated recruitment procedures at University of Alabama at Birmingham: Added breast research specialists who are

		embedded within the breast cancer clinical team
4	1/23/20	Modified recruitment strategy: Included self-referral as a result of bringing recruitment brochures to wellness fairs and cancer survivorship events
5	2/18/20	Adjusted screening tool: Work and Social Adjustment Scale modified to attribute challenges to “cancer” versus “health”<br>Modified recruitment strategy: Included advertising the study on social media
6	4/24/20	Change to measure: Add 3 questions about COVID-19 status
7	1/20/21	Change to data collection: Added option to send link to an electronic version of surveys or mail a hard copy and postage paid envelope and identified points at which we would stop attempting to contact participants for each assessment
8	11/2/21	Change named principal investigator due to Dr. Lyons changing employment
9	1/11/23	Change to data collection: Moved data collection activities to Dr. Lyons’ new institution<br>Updated data safety monitoring plan to conform to Dr. Lyons’ new institution policies<br>Change participant reimbursement to come from Dr. Lyons’ new institution<br>Updated data sharing policies between three sites (Massachusetts General Hospital, Dartmouth-Hitchcock and University of Alabama at Birmingham
