# Supplementary material for: Telephone-Based Rehabilitation Intervention to Optimize Activity Participation After Breast Cancer: A Randomized Clinical Trial
Source: JAMA Netw Open. 2024 Mar 22;7(3):e242478. doi: 10.1001/jamanetworkopen.2024.2478 (PMC10960198; doi:10.1001/jamanetworkopen.2024.2478)
Supplement: Supplement 2. — eTable. Association Between Participant Characteristics and Missing Data at Follow-Up [file jamanetwopen-e242478-s002.pdf]

Supplemental Online Content

Lyons KD, Wechsler SB, Ejem DB, et al. Telephone-based rehabilitation intervention to optimize activity participation after breast cancer: a randomized clinical trial. *JAMA Netw Open*. 2024;7(3): e242478. doi:10.1001/jamanetworkopen.2024.2478

eTable. Association Between Participant Characteristics and Missing Data at Follow-Up

© 2024 Lyons KD et al. *JAMA Network Open*.

eTable. Association Between Participant Characteristics and Missing Data at Follow-Up

Characteristic	Follow-up Data Collection				Effect size	P
	Complete (n=210)		Incomplete (n=74)			
	n	(%)	n	(%)	V or d	
Study group					0.04	0.495
BA/PS	109	(51.9)	35	(47.3)		
Attention Control	101	(48.1)	39	(52.7)		
Age, M (SD)	57.0	(10.1)	53.4	(10.3)	d=0.35	0.010
Site						
Dartmouth Clinic	58	(26.6)	13	(17.6)	0.18	0.011
Facebook	119	(56.7)	38	(51.4)		
UAB Clinic	33	(15.1)	23	(31.1)		
Race					0.13	0.104
Black/African American	29	(13.8)	18	(24.3)		
White/Caucasian	179	(85.2)	55	(74.3)		
Other	2	(1)	1	(1.4)		
Ethnicity					0.09	0.142
Hispanic/Latino	6	(2.9)	0	(0)		
Not Hispanic/Latino	204	(97.1)	74	(100)		
Employment Status					0.18	0.063
Employed (FT/PT)	113	(54.1)	39	(53.4)		
Homemaker/Retired	62	(29.7)	15	(20.5)		
On Short/Long Term Disability	15	(7.2)	10	(13.7)		
Unemployed	13	(6.2)	9	(12.3)		
Other	6	(2.9)	0	(0)		
Education Level					0.11	0.161
Some High school/High school Graduate	16	(7.6)	11	(15.3)		
Some College (2 year)	49	(23.3)	16	(22.2)		
Graduate (4 year, Master's, Doctoral)	145	(69)	45	(69)		
Marital Status					0.09	0.478
Never Married	14	(6.7)	5	(6.8)		
Married/Living with a partner	143	(68.1)	44	(60.3)		
Separated/Divorced	46	(21.9)	19	(26)		
Widowed	7	(3.3)	5	(6.8)		
Number of Dependent Children Living at Home					0.14	0.230
0	152	(72.4)	46	(63)		
1	31	(14.8)	11	(15.1)		
2	21	(10)	10	(13.7)		
3	5	(2.4)	4	(5.5)		
4	1	(0.5)	2	(2.7)		

© 2024 Lyons KD et al. *JAMA Network Open*.

Characteristic	Follow-up Data Collection Complete (n=210) n	(%)	Incomplete (n=74) n	(%)	Effect size V or d	P
Insurance Status					0.13	0.192
Other/None, Self pay	7	(3.3)	5	(6.8)		
Medicaid/Medicare, or other government insurance	60	(28.6)	18	(24.3)		
Private through employer	136	(64.8)	45	(60.8)		
Self-Purchased	7	(3.3)	6	(8.1)		
Household Income					0.11	0.206
Less than $40,000 per year	40	(19)	20	(27.4)		
$40,000 per year or more	167	(79.5)	53	(72.6)		
Rurality						
Urban	126	(62.4)	55	(76.4)	0.13	0.031
Rural	76	(37.6)	17	(23.6)		
Cancer Stage					0.18	0.009
Stage I	98	(46.7)	21	(28.4)		
Stage II	73	(34.8)	40	(54.1)		
Stage III	39	(18.6)	13	(17.6)		
Cancer Treatment						
Surgery	210	(100)	72	(97.3)	0.14	0.017
Radiation	161	(76.7)	57	(77)	0.00	0.950
Chemotherapy	137	(65.2)	49	(66.2)	0.01	0.879
Time Since End of Primary Treatment					0.08	0.222
<6 months	125	(59.5)	36	(48.6)		
>6 months	85	(40.5)	38	(51.4)		
	**Mean**	**(SD)**	**Mean**	**(SD)**	**Cohen's d**	**P**
PROMIS: Satisfaction with Social Roles & Activities:						
T score	46.0	(6.5)	44.9	(7.2)	0.17	0.22
PROMIS: Ability to Participate in Social Roles & Activities:						
T score	43.5	(5.3)	42.9	(5.6)	0.11	0.405
Work Limitations Questionnaire scale score[a]	35.8	(19)	33.7	(16.4)	0.12	0.528
Quality of Life, FACT-G:						
Physical	17.5	(5.1)	15.6	(5.6)	0.37	0.007
Social	20.1	(5.5)	17.9	(6.6)	0.38	0.006
Emotional	16.1	(4.2)	15	(5.4)	0.24	0.073
Functional	16.8	(4.8)	15.1	(6.2)	0.32	0.019
Overall score	70.5	(15.5)	63.6	(19.7)	0.41	0.003

© 2024 Lyons KD et al. *JAMA Network Open*.

Characteristic	Follow-up Data Collection				Effect size	P
	Complete (n=210)		Incomplete (n=74)			
	Mean	(SD)	Mean	(SD)	Cohen's d	
Coping:						
Brief COPE: Active coping	6.4	(1.6)	6.4	(1.5)	0.02	0.895
Brief COPE: Planning	6.4	(1.7)	6.4	(1.7)	0.03	0.805
Brief COPE: Positive reframing	6.4	(1.7)	6.4	(1.7)	0.01	0.936
Goal Adjustment Scale:						
Disengagement score	10.4	(3.2)	10.5	(3.1)	0.01	0.972
Reengagement score	21.3	(3.9)	21.4	(3.8)	0.04	0.773
Hospital Anxiety Depression Scale (HADS):						
HADS Anxiety score	9.0	(4.2)	9.6	(4.8)	0.13	0.337
HADS Depression score	6.0	(3.5)	7.2	(4.5)	0.3	0.026
Work and Social Adjustment Scale (WASA)	16.6	(6.7)	17.8	(7.3)	0.18	0.187

a: Only currently employed (n=152)

© 2024 Lyons KD et al. *JAMA Network Open*.
