# Supplementary material for: Telephone-Based Rehabilitation Intervention to Optimize Activity Participation After Breast Cancer: A Randomized Clinical Trial
Source: JAMA Netw Open. 2024 Mar 22;7(3):e242478. doi: 10.1001/jamanetworkopen.2024.2478 (PMC10960198; doi:10.1001/jamanetworkopen.2024.2478)
Supplement: Supplement 3. — Data Sharing Statement [file jamanetwopen-e242478-s003.pdf]

Data Sharing Statement

Lyons. Telephone-Based Rehabilitation Intervention to Optimize Activity Participation After Breast Cancer. *JAMA Netw Open*. Published March 22, 2024.
doi:10.1001/jamanetworkopen.2024.2478

Data
Data available: Yes
Data types: Deidentified participant data, Data dictionary
How to access data: Available upon request to PI at KLyons2@mghihp.edu
When available: With publication

Supporting Documents
Document types: None

Additional Information
Who can access the data: Researchers whose proposed use of the data has been approved
Types of analyses: Secondary analyses
Mechanisms of data availability: With a signed data access agreement
